# Machine learning-based identification and brain network characterization of flight cadets compared with air traffic control (ATC) students using static and dynamic functional connectivity

**DOI:** 10.3389/fnhum.2026.1873697

**Published:** 2026-08-25

**Authors:** Lu Ye, Yang Zhang, Liya Ba, Dongfeng Yan

**Affiliations:** 1 Flight Technology College, Civil Aviation Flight University of China, Guanhan, Sichuan, China; 2 College of General Aviation and Flight, Nanjing University of Aeronautics and Astronautics, Nanjing, China; 3 Sichuan Provincial Engineering Research Center of Domestic Civil Aircraft Flight and Operation Support, Guanhan, Sichuan, China

**Keywords:** dynamic functional connectivity, flight cadets, machine learning, resting-state functional magnetic resonance imaging, static functional connectivity

## Abstract

**Introduction:**

This study used ATC students with a comparable civil aviation educational background and aviation-related cognitive demands, but no actual flight experience, as the control group. This study aimed to systematically compare the performance of static and dynamic functional connectivity in distinguishing flight cadets and to explore time-varying brain network characteristics associated with flight training.

**Methods:**

Resting-state functional magnetic resonance imaging (rs-fMRI) data were collected from 39 flight cadets and 37 ATC students, from which static functional connectivity (sFC) and dynamic functional connectivity (dFC) features were extracted. Following feature selection, five classifiers, including Support Vector Machine (SVM), eXtreme Gradient Boosting (XGBoost), Random Forest (RF), Gaussian Naive Bayes (GNB), and K-Nearest Neighbors (KNN), were systematically evaluated.

**Results:**

The primary analysis showed that dFC generally achieved better classification performance than sFC. The optimal dFC-SVM model reached an Area Under the ROC Curve (AUC) of 0.949, with an accuracy of 0.895, a sensitivity of 0.945, and a specificity of 0.848, whereas the best sFC model achieved an AUC of only 0.828. Under a more stringent supplementary validation procedure, the dFC-SVM and sFC-SVM models achieved AUCs of 0.65 and 0.59, respectively. The window-length sensitivity analysis showed that model performance remained within a similar range across the tested window lengths. High-contribution connections identified in the primary analysis involved brain regions such as the left insula and parahippocampal gyrus and were distributed across the Default Mode Network (DMN), Central Executive Network (CEN), and Sensorimotor Network (SMN).

**Discussion:**

Taken together, dFC may contain additional time-varying discriminative information beyond static connectivity. However, the magnitude of this advantage was limited in the supplementary validation, and the related findings should be regarded as preliminary evidence requiring further validation. By adopting a comparable control design within the aviation domain, this study further characterized neurofunctional representations associated with flight training experience and provided neuroimaging clues for subsequent longitudinal validation incorporating training stage, behavioral performance, and flight performance, as well as for exploring adaptive monitoring of flight training and multidimensional auxiliary assessments.

## Introduction

In recent years, the rapid development of the global civil aviation industry has promoted air transport as an important driver of economic growth, while simultaneously imposing higher demands on flight safety. At present, human factors have become one of the major contributors to aviation accidents (Oraith et al., 2021; Masi et al., 2023), further underscoring the importance of pilot selection, screening, and training in enhancing flight safety (Kelly and Efthymiou, 2019). As a distinct occupational group, pilots undergo a rigorous and standardized process of selection and training (Tian et al., 2022). Pilot training is characterized by a long duration, high cost, and sustained pressure arising from regular assessments and attrition throughout the entire process. As the first step toward a professional piloting career, flight cadets must complete a series of demanding training courses designed to strengthen both operational skills and cognitive abilities under complex conditions, thereby enabling them to respond effectively to a wide range of unexpected situations (Chen et al., 2023). The communication and expressive abilities, learning adaptability, and decision-making capacity of flight cadets are all closely related to the functional mechanisms of the brain. Investigating the neural functional mechanisms associated with flight cadets’ performance of flight tasks is not only of great significance for the safe operation of aircraft, but may also provide objective physiological parameters for the assessment and evaluation of flight cadets, thereby meeting the requirements proposed by the Civil Aviation Administration for pilot training reform (Civil Aviation Administration of China, 2020).

Resting-state functional magnetic resonance imaging (rs-fMRI) explores spontaneous neural activity in the brain under task-free conditions by detecting changes in the blood oxygen level-dependent signal (Chen et al., 2021; Fallahi et al., 2021). rs-fMRI can reflect functional coordination among brain networks (Honey et al., 2009; Eickhoff et al., 2016), thereby revealing associations between brain function and vigilance (Shen et al., 2016), working memory (Constantinidis and Klingberg, 2016), and psychomotor ability (Chen et al., 2024), and has now been widely applied in neuroimaging research. Previous studies using rs-fMRI have shown that flight tasks can alter the structural and functional patterns of the pilot brain. Xu et al. (2023) found that, relative to healthy controls, pilots exhibited significant differences in the left cuneus and right cerebellum, with markedly increased spontaneous activity in brain regions related to visual information processing and bodily coordination. Chen et al. (2020) reported increased functional connectivity among the Central Executive Network (CEN), Default Mode Network (DMN), and Salience Network (SN), together with decreased functional connectivity within the CEN, which may suggest greater cognitive flexibility in pilots than in the general population. Radstake et al. (2023), by comparing functional connectivity data between pilots and non-pilots, found alterations in vestibular, motor, and multisensory processing in the pilot brain.

In recent years, machine learning (ML), owing to its data-driven nature, has shown substantial potential in brain signal analysis and neural state recognition by enabling the extraction of discriminative features from high-dimensional neurophysiological data (Pereira et al., 2009). Recent electroencephalography (EEG)-based studies have further shown that deep learning frameworks designed to capture the spatiotemporal characteristics of brain signals can effectively support the automated recognition of complex psychological and operational states. Geda et al. (2026) proposed an end-to-end deep learning framework, EmoDLNet, which integrates two-dimensional and one-dimensional convolutional neural networks with hierarchical gated recurrent units to automatically extract multiscale spatiotemporal features from raw EEG signals for cross-subject emotion recognition. For pilot fatigue detection, Chen et al. (2026) combined brain power maps with a hybrid CNN-BiMGRU-MHAM architecture and employed an improved sparrow search algorithm to optimize key hyperparameters, thereby enabling the automated recognition of different fatigue states during simulated flight. These studies suggest that jointly modeling the spatial distribution, temporal dependencies, and task-specific information of brain signals can provide an effective methodological pathway for the objective recognition of complex cognitive states and aviation operational states. In rs-fMRI studies, models such as Support Vector Machine (SVM), eXtreme Gradient Boosting (XGBoost), Random Forest (RF), Gaussian Naive Bayes (GNB) and K-Nearest Neighbors (KNN) have been widely applied in brain network research and have played important roles in the analysis of various neurological disorders, including obsessive-compulsive disorder (Liu et al., 2021), Alzheimer’s disease (Venugopalan et al., 2021), and depression (Yan et al., 2020). Functional Connectivity (FC), as one of the core metrics derived from rs-fMRI, can be used to quantitatively characterize the functional relationships between different brain regions (Friston, 2011). According to the assumption regarding the temporal stability of functional brain networks, FC can be divided into static Functional Connectivity (sFC) and dynamic Functional Connectivity (dFC). sFC assumes that functional brain networks remain stable over a certain period and calculates the temporal correlations between different brain regions on the basis of regional Blood Oxygenation Level-Dependent (BOLD) signals (Jie et al., 2018). Previous studies have shown that sFC can serve as an effective feature for identifying flight cadets (Ye et al., 2026). However, with the deepening of research, accumulating evidence has indicated that even in the resting state, functional brain activity is not fixed, but instead exhibits marked dynamic characteristics (Tu et al., 2019). Against this background, the concept of dFC was proposed, revealing the fluctuation and reorganization characteristics of functional brain networks across different time periods and providing richer information. The sliding time-window approach is currently the most commonly used method for investigating dFC. Its basic principle is to divide the complete time series into multiple temporal windows with fixed window width and step size, and then to calculate FC strength within each window separately, thereby examining the dynamic changes in FC across adjacent time windows.

By analyzing the temporal correlations between two target brain regions, FC can capture subtle variations in brain activity within high-dimensional features. FC is often combined with ML to efficiently identify lesion-related brain regions in patients, thereby enabling timely diagnosis and intervention. For example, Yan et al. (2020) investigated patterns of functional connectivity in patients with major depressive disorder and healthy controls, extracted sFC and dFC metrics as features, and performed classification using SVM. Their results showed that dFC achieved better discriminative performance than sFC, with an Area Under the Curve (AUC) of 0.9913, providing an important reference for the quantitative identification of patients with major depressive disorder. Sun et al. (2020) collected functional magnetic resonance imaging (fMRI) data from hyperactive patients with attention deficits and normally developing controls, and used interregional functional connectivity as classification features. The study showed that, after applying SVM with Leave-One-Out Cross-Validation (LOOCV), the highest classification accuracy was achieved, correctly identifying 85.3% of the participants. Moreover, most of the most discriminative FC features were primarily concentrated in the cerebellum, DMN, and frontoparietal regions.

Existing machine learning studies of brain functional connectivity have focused primarily on neuropsychiatric disorders, with relatively limited attention paid to healthy occupational groups such as flight cadets. Previous studies have shown that sFC can serve as an effective feature for identifying flight cadets. However, flight tasks involve complex dynamic cognitive demands, including continuously changing information and ongoing adjustments in operational requirements. Their neural basis may therefore be reflected not only in the average connectivity strength between brain regions, but also in the flexible, time-varying coordination among multiple brain networks. dFC may provide richer information for capturing such neurofunctional characteristics; however, the performance of sFC and dFC in distinguishing flight cadets from air traffic control (ATC) students with comparable civil aviation educational backgrounds and aviation-related cognitive demands has not been systematically compared. Accordingly, the present study separately extracted sFC and dFC features from flight cadets and ATC students and constructed classification models using five algorithms: SVM, XGBoost, RF, GNB, and KNN. Interpretability analyses were further performed to identify functional connections with relatively high discriminative contributions and characterize their distribution across brain networks, thereby comparing the classification performance of static and dynamic connectivity features. To further evaluate the dependence of the comparative findings on the feature-processing pipeline and dFC window-parameter settings, a more stringent supplementary validation procedure was used to recompare sFC-SVM and dFC-SVM, and sensitivity analyses across different sliding-window lengths were conducted to assess the sensitivity of dFC-SVM classification performance to the choice of window length. On this basis, the study further investigated dynamic brain network characteristics associated with flight training experience, with the aim of providing candidate neuroimaging clues for future research on adaptation to flight training and personalized auxiliary assessment.

## Materials and methods

### Participants

Participants were recruited from the Civil Aviation Flight University of China. The inclusion criteria for all participants were as follows: (1) male; (2) aged 21–25 years; (3) right-handed; (4) no metallic implants in the body; (5) physically healthy at the time of the experiment; (6) holder of a bachelor’s degree in engineering; (7) no history of psychiatric disorders; (8) no drug or alcohol dependence; (9) no history of traumatic brain injury, cerebrovascular disease, or chronic pain; and (10) fully informed about the experimental procedures, willing to participate voluntarily, and having signed written informed consent before the experiment. It should be noted that, within the recruitment sources available for the present study, the number of female flight cadets who met the same training-stage, licensing, and aeromedical fitness requirements and were also eligible to undergo MRI scanning was extremely limited. Consequently, it was not feasible to establish a female flight-cadet subgroup that was comparable to the male flight cadets in terms of sample size and training background. Sex may have potential effects on brain structure, functional connectivity patterns, and certain cognitive-behavioral measures (Cahill, 2006). Therefore, including only a tiny number of female participants in the present study could have increased within-group heterogeneity and introduced potential confounding between sex-related effects and flight-training experience.

All flight cadets in the experimental group had completed the standardized integrated training curriculum approved by the Civil Aviation Administration of China (CAAC), and all had obtained a private pilot license, instrument rating, and commercial pilot license. Although they were still institutionally classified as “flight cadets,” their possession of CAAC-certified pilot licenses and qualification for solo flight indicates that, in terms of skill level, they had effectively reached the standard of junior experienced pilots rather than remaining at the novice stage. For the sake of terminological accuracy and consistency, however, they are uniformly referred to as “flight cadets” throughout the present study. All cadets had completed no fewer than the 235 flight training hours required by the university. These training hours were highly structured and stage-specific, including basic single-engine aircraft handling and visual navigation training under visual meteorological conditions; complex procedural flight and attitude control training under simulated and actual instrument meteorological conditions; and advanced commercial flight operations in multi-engine aircraft. Importantly, these 235 h explicitly included the solo-flight phase in which cadets operated as pilot-in-command, as well as night-flight operations characterized by high cognitive workload demands. All flight cadets strictly followed a unified standardized training syllabus, passed all required assessments, and were at the critical stage immediately preceding advanced trainer aircraft training, thereby showing a highly homogeneous training background and skill level.

The control group consisted of fourth-year undergraduate students majoring in air traffic control (ATC). These students had completed all theoretical courses and basic professional practical training required by the curriculum, had satisfied the academic requirements for graduation, and were in the final stage before graduation. The selection of ATC students as the control group was based on three main considerations. First, under the civil aviation recruitment system in China, both ATC and flight technology are undergraduate programs with a clear profession-oriented focus. Students in both majors are required to meet the first-tier undergraduate admission standards of their respective provinces and undergo psychological aptitude assessments related to civil aviation occupational adaptability at the early stage of enrollment. Second, participants in both groups were full-time undergraduates from the same university, had the same years of education, and had received long-term standardized, procedure-based, and safety-oriented professional education in civil aviation. More importantly, from the perspective of the civil aviation operational system, pilots and air traffic controllers jointly constitute the core components of air–ground coordination. Although their specific occupational responsibilities differ, both operate in complex environments characterized by stringent safety requirements, rapidly changing information, and high decision-making demands. These roles require continuous monitoring of multisource information, allocation of attentional resources, execution of standard operating procedures, and goal-directed judgments and decisions under time pressure. In this regard, ATC students are more comparable to flight cadets in terms of operational context and cognitive task characteristics. Therefore, the use of ATC students as the control group was intended to control, as far as possible, for general civil aviation educational background and aviation-related cognitive demands, thereby enabling a more targeted investigation of brain functional characteristics associated with flight training.

The selection, training, and stage-specific assessment of flight cadets are guided by explicit industry regulations and evaluation criteria. According to the Rules on Medical Standards and Certification for Civil Aviation Personnel of China (CCAR-67), student pilots trained toward airline transport pilot qualifications or commercial pilot qualifications for airplanes and rotorcraft are required to hold a Class I medical certificate when applying for a license or exercising the privileges of the corresponding license. The validity period of a Class I medical certificate is generally 12 months; therefore, flight cadets must undergo continuous aeromedical evaluation to maintain its validity. Air traffic control personnel are required to hold a Class III medical certificate, which also has a prescribed validity period. In the present study, the two groups of students met the respective aeromedical standards for flight personnel and air traffic control personnel. Flight cadets are further required to satisfy the training and assessment requirements related to occupational competence within the Chinese civil aviation flight training system. According to relevant documents issued by the Civil Aviation Administration of China concerning the comprehensive reform of flight training in air transport airlines and the development of a life-cycle management system for pilot skills, current pilot training and evaluation place particular emphasis on a competency-based and evidence-based training framework across the full professional life cycle (Civil Aviation Administration of China, 2020). The flight cadets included in the present study had not only completed the required flight hours and stage-specific training but had also passed the standardized assessments mandated by these requirements.

It should be particularly noted that none of the control participants held any category of pilot license, nor did they have any simulator or actual flight experience. After careful screening, data from a total of 76 participants were ultimately included in the study, comprising 39 flight cadets and 37 controls from ground-specialty programs. This study was conducted in accordance with the Declaration of Helsinki and was approved by the Ethics and Human Protection Committee of the Magnetic Resonance Imaging Research Center at the University of Electronic Science and Technology of China (approval number: 1420200408-07). The present study used the same rs-fMRI dataset as a previous dFNC study conducted by our research group (Ye et al., 2025). The previous study focused on dynamic connectivity among the triple networks and its associations with behavioral performance, whereas the present study further performed machine learning-based comparative and interpretability analyses using whole-brain sFC and dFC features derived from the AAL atlas.

### Data acquisition

Magnetic resonance imaging data were acquired at the Magnetic Resonance Imaging Research Center of the University of Electronic Science and Technology of China, Sichuan Province, using a GE Discovery MR750 3.0 T magnetic resonance imaging scanner equipped with an 8-channel phased-array head coil. Structural magnetic resonance imaging data were obtained using a three-dimensional spoiled gradient-recalled echo sequence with the following parameters: repetition time (TR) = 5.952 ms, echo time (TE) = 1.964 ms, flip angle = 9°, acquisition matrix = 256 × 256, field of view (FOV) = 256 mm × 256 mm, slice thickness = 1.0 mm, and 154 slices. Functional magnetic resonance imaging data were acquired using a standard gradient-echo pulse sequence with the following parameters: TR = 2000 ms, TE = 30 ms, flip angle = 90°, acquisition matrix = 64 × 64, FOV = 240 mm × 240 mm, slice thickness = 4.0 mm with an interslice gap of 0.4 mm, 35 slices, a scan duration of 510 s, and a total of 255 volumes. Before scanning, all participants were instructed to remove all metallic objects and wear noise-reducing earplugs. During the scan, they were required to keep their eyes closed, remain awake throughout the procedure, and refrain from thinking about any specific topic. To minimize head motion, foam pads were placed on both sides of the head to stabilize head position.

### Data processing

Preprocessing of the rs-fMRI data was performed using RESTplus software based on the MATLAB 2022b platform.^1^ The preprocessing steps were as follows: (1) the first five time points were discarded to reduce the influence of magnetic field instability; (2) slice-timing correction was performed to minimize differences in acquisition time across slices; (3) head-motion correction was conducted, and samples with head translation >2 mm or rotation >2° were excluded (Jenkinson et al., 2002); (4) spatial normalization was performed by first coregistering the individual functional magnetic resonance images to the structural magnetic resonance images, followed by nonlinear transformation into the Montreal Neurological Institute (MNI) space and resampling to 3 mm × 3 mm × 3 mm; (5) spatial smoothing was applied using a Gaussian kernel with a radius of 6 mm; (6) linear drift was removed; (7) nuisance covariates were regressed out, including white matter, head motion, and cerebrospinal fluid signals; and (8) temporal filtering was performed to retain signals within the range of 0.01–0.08 Hz.

The sFC metrics were calculated using RESTplus. Ninety brain regions from the Automated Anatomical Labeling (AAL) atlas (Tzourio-Mazoyer et al., 2002) were selected as Regions of Interest (ROIs). First, all voxels within each ROI were averaged to obtain the corresponding time series. Next, Pearson correlation coefficients were calculated pairwise between all ROIs to quantify the correlations in mean signal intensity between different brain regions. This procedure yielded a symmetric 90 × 90 matrix, with all diagonal elements equal to 1 because each brain region was perfectly correlated with itself. The resulting correlation matrix was then transformed using Fisher’s *Z* transformation. Owing to the symmetry of the matrix, only the elements in the upper triangular portion were extracted as sFC features. Accordingly, the number of features contained in each sFC matrix was: $\frac{\left[90\times (90-1)\right]}{2}=4005$ , yielding a total of 304,380 sFC features across all 76 participants.

dFC analysis was performed using DynamicBC software.^2^ A sliding time-window approach was used to segment the 250 time points. Considering the trade-off between temporal resolution and the stability of within-window functional connectivity estimates, and referring to the commonly used window range of 30–60 s in previous studies (Shirer et al., 2012; Hutchison et al., 2013), the window length was set to 30 TR (60 s), with a sliding step of 1 TR (2 s). Continuous overlapping fixed rectangular windows were used, with adjacent windows overlapping by 29 TR and no temporal gaps between windows. Based on the AAL atlas, the brain was divided into 90 distinct functional regions, and FC matrices were calculated among the 90 ROIs. After the sliding-window analysis, 221 symmetric dFC matrices were obtained for each participant. Each dFC matrix contained 90 × 90 elements, and only the upper triangular elements of the symmetric matrix were extracted. Ultimately, a total of 76 × 221 × 4,005 dFC features were obtained.

In the present study, a two-sample *t*-test was used for feature selection. The two-sample *t*-test can be used to evaluate the degree of difference in each feature between different classes (e.g., positive and negative classes), and it has been widely applied for feature selection in ML-based neuroimaging studies with good performance (Yan et al., 2020). For the extracted sFC and dFC features, the present study used a two-sample t-test to compare mean differences between positive and negative samples, with statistical significance determined on the basis of the *p* value. Subsequently, different feature subsets were selected according to predefined thresholds, thereby retaining the most discriminative features. The feature-selection algorithm based on the two-sample *t*-test was implemented in Scipy.

### Model construction and performance evaluation

After feature selection using the two-sample *t*-test, the sFC and dFC metrics were used as input features for the construction of classification models. All classification models were implemented in Python based on the scikit-learn library, and five classification methods were employed, including SVM, XGBoost, RF, GNB, and KNN. To optimize model performance, the hyperparameters of the classification algorithms were tuned using a grid search approach. The dataset was randomly divided into a training set and a test set at a ratio of 8:2 to evaluate the generalization ability and classification performance of the models.

Accuracy, Sensitivity, Specificity, the Receiver Operating Characteristic (ROC) curve and its Area Under the Curve (AUC), and the confusion matrix were used as evaluation metrics. To accurately assess model generalizability and obtain stable and reliable performance estimates, the present study adopted a nested evaluation strategy combining repeated random splitting with Leave-One-Out Cross-Validation (LOOCV). The entire evaluation procedure was repeated for 50 iterations. In each iteration, the original dataset was randomly divided into a training set and an independent test set at a ratio of 8:2. During model training and hyperparameter optimization, LOOCV was introduced within the training set to maximize data utilization under the small-sample condition. Combined with a grid search strategy, the average score obtained from LOOCV was used to determine the optimal hyperparameter combination for the current training set. The model was then refitted on the full training set using this optimal parameter combination and finally evaluated on the test set, which had not participated in model training. By aggregating the test results across the 50 iterations, the mean values of all performance metrics were calculated. The ROC curve was used to quantify model performance, and performance was further evaluated using the mean AUC obtained above together with analysis of the confusion matrix. To assess the statistical reliability of model prediction performance, a permutation test was performed. Specifically, the class labels of the training data were first randomly shuffled, and cross-validation was then repeatedly conducted on the permuted dataset. This procedure was repeated 10,000 times to calculate the generalization performance of the classifier under random labels. Classification performance was considered reliable when the generalization performance of the classifier trained on the true class labels exceeded the 95% confidence interval of the classifiers trained on randomly permuted class labels.

**Figure 1:**
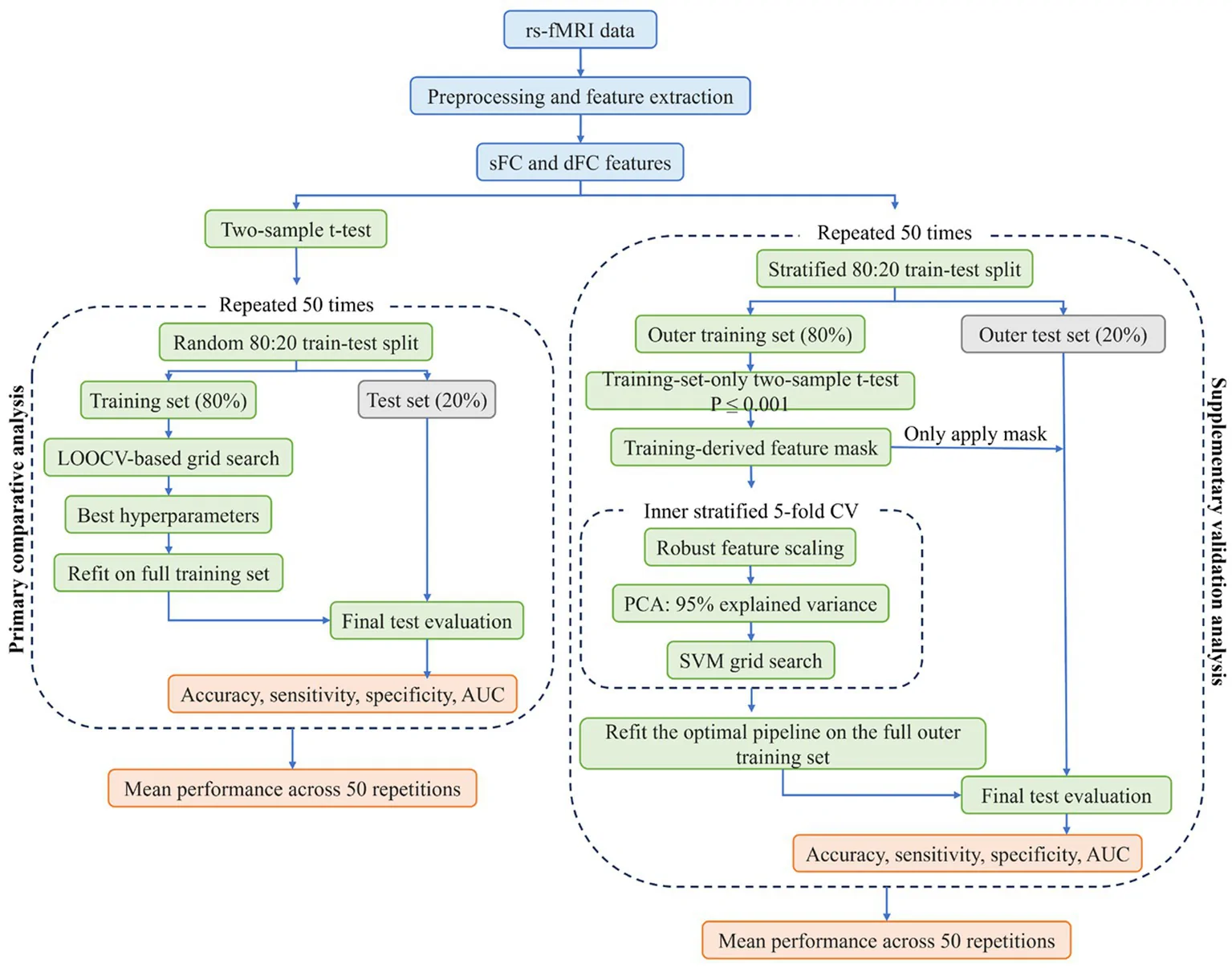
Overview of the machine learning analysis workflow.

To assess the robustness of the primary comparative findings to the timing of feature selection and the strategy used to process high-dimensional features, supplementary validation analyses were conducted for the sFC-SVM and dFC-SVM models. The analysis used 50 repeated stratified random splits. In each repetition, all participants were first divided into an outer training set and an independent test set using an 80:20 ratio. A two-sample *t*-test was then performed using only the training data and their class labels, with *p* ≤ 0.001 used as the feature-selection threshold. The feature mask determined from the training set was directly applied to the corresponding test set, ensuring that the test set was not involved in feature selection. Robust scaling was applied to the features retained after the *t*-test to reduce the effects of outliers and differences in feature scale. Principal component analysis (PCA) was subsequently performed, retaining the components that collectively explained 95% of the cumulative variance, which were then entered into the SVM classifier. During stratified five-fold cross-validation within the outer training set, the scaling parameters and PCA components were estimated exclusively from each inner training fold and then applied to the corresponding validation fold. The SVM kernel and associated hyperparameters were optimised using grid search, and the optimal parameter combination was selected according to the mean performance across the five folds. The final SVM model was then trained on the complete outer training set and evaluated on the independent outer test set. Finally, the results from the 50 repeated analyses were aggregated, and model performance was expressed as the mean across repetitions. An overview of the machine learning evaluation workflow is presented in Figure 1.

To further evaluate the sensitivity of dFC classification performance to the selected sliding-window length, dFC features were additionally recalculated using window lengths of 20 TR and 40 TR. All three window lengths, including the original 30-TR window, used continuous overlapping fixed rectangular windows with a sliding step of 1 TR. Under the 20-TR and 40-TR conditions, adjacent windows overlapped by 19 and 39 TR, respectively, with no temporal gaps between windows. Given 250 valid time points, the 20-TR and 40-TR windows yielded 231 and 211 temporal windows, respectively. The same data-processing procedure was applied across all window-length conditions. Because SVM was the best-performing dFC classifier in the primary analysis and was also used to compare sFC and dFC in the supplementary validation, the window-length sensitivity analysis focused on the dFC-SVM model. For each of the three window lengths, the same evaluation procedure as that used in the supplementary validation was applied. Specifically, 50 repeated stratified 80:20 train-test splits were performed, and a two-sample t-test was conducted exclusively within each outer training set using *p* ≤ 0.001 as the feature-selection threshold. Robust scaling, PCA retaining components that collectively explained 95% of the cumulative variance, and SVM hyperparameter optimization were all embedded within stratified five-fold cross-validation in the outer training set. Finally, the mean accuracy, sensitivity, specificity, and AUC obtained from the independent test sets were summarized for the three window-length conditions.

To further investigate the important functional brain connections that distinguish the flight cadet group from the control group from the perspective of feature contribution, the present study introduced SHapley Additive exPlanations (SHAP) analysis based on the optimal classification model, SVM. SHAP is an advanced attribution technique grounded in cooperative game theory. It can fairly and consistently allocate the predictive output of a model to each input feature, thereby quantifying the contribution of each feature to the prediction for an individual sample as well as to the overall model output, and providing a transparent interpretation of the decision-making process of black-box models (Meng et al., 2021).

## Results

### Demographic results

A total of 76 participants were ultimately included in the present study, comprising 39 flight cadets in the flight group and 37 ATC students in the control group. As shown in Table 1, the two groups were completely matched in terms of sex (specifically biological sex), handedness, and years of education. All flight cadets had completed the standardized training curriculum approved by the CAAC and had accumulated an average of 240.69 ± 8.61 flight hours. With regard to age, the results of the independent-samples t-test showed that the mean age of the flight group was slightly higher than that of the control group, and the difference was statistically significant. Although a statistically significant age difference was observed between the two groups, the actual difference in mean age was very small (less than 0.5 years), and all included participants were strictly restricted to the narrow physiological developmental window of 21–25 years. Previous studies in developmental cognitive neuroscience have shown that, during this stage of young adulthood, the large-scale topological organization of brain networks and intrinsic functional connectivity patterns have reached a highly mature and relatively stable state (Dosenbach et al., 2010). The present study therefore considered the potential biological interference of such subtle age variation on functional brain connectivity to be extremely limited. During the extraction of individual-level FC features and their input into the machine learning models, no additional covariate regression for age was performed in order to preserve the integrity of the original brain network topology to the greatest extent possible.

**Table 1:** Demographic characteristics of the study participants.

Participant characteristics	Flight cadets (*N* = 39)		Controls (*N* = 37)		*t*-value	*p*-value
	Mean	SD	Mean	SD		
Age (years)	22.85	0.96	22.38	0.92	2.16	0.03
Sex (male/female)	39/0	0	37/0	0	–	–
Education (years)	16	0	16	0	–	–
Right-handedness (%)	100	0	100	0	–	–
Flight hours	240.69	8.61	0	0	–	–

### Feature selection results

In the study, two types of metrics, sFC and dFC, were extracted. When performing the two-sample t-test on different metrics, the appropriate selection of thresholds was crucial for the effectiveness of feature screening. Through multiple comparative analyses, the optimal threshold for each metric was determined. Table 2 presents the optimal threshold level for each metric type and the corresponding number of features retained after screening.

**Table 2:** Threshold levels and the number of selected features.

Feature set	sFC	dFC
*p*-value	0.01	0.005
Number of features	28	62

### Comparison of the performance of different classifier models

**Figure 2:**
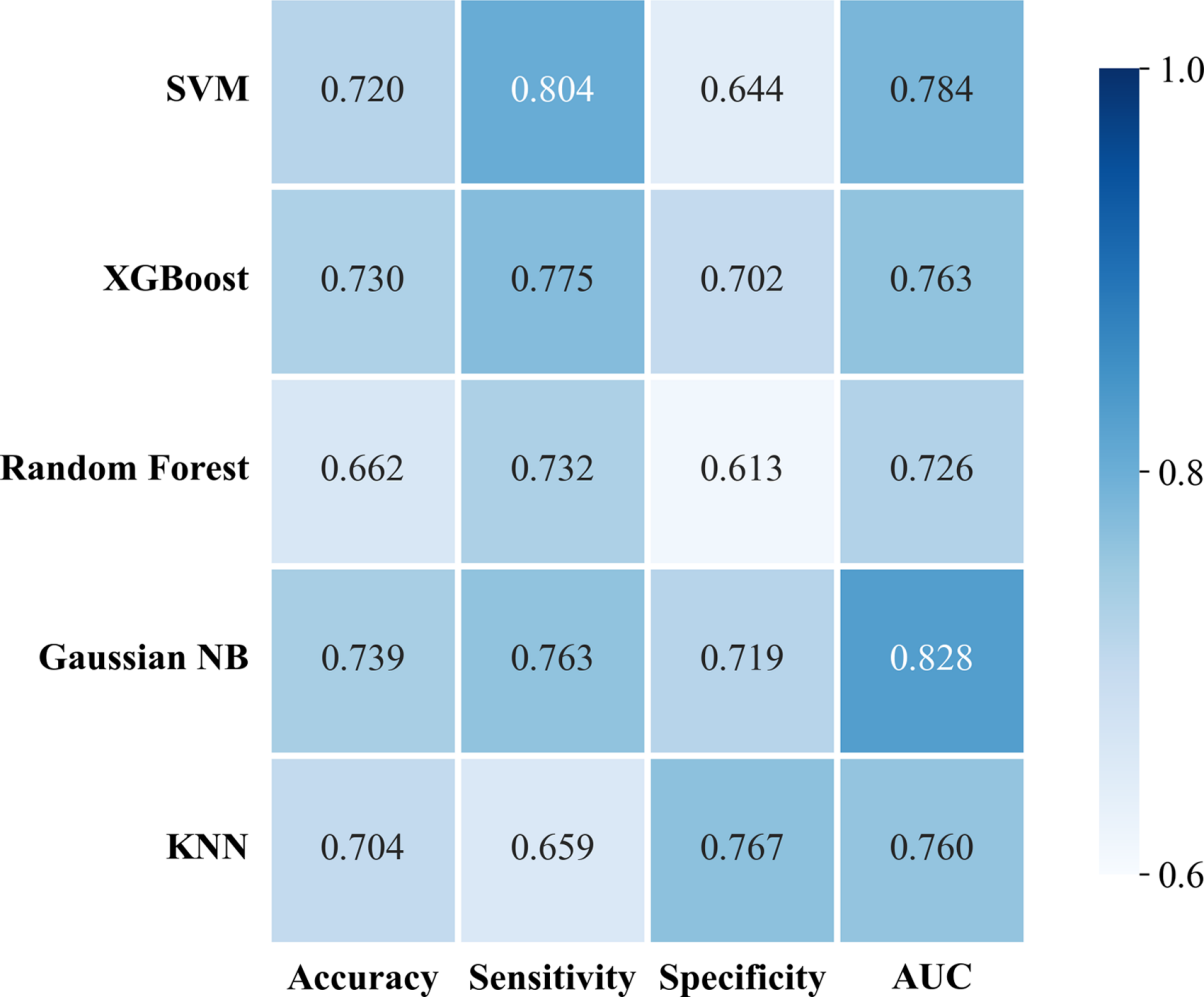
Performance evaluation metrics of different models based on sFC.

The classification results using sFC as features are shown in Figure 2. In Figure 2, the rows correspond to different machine learning algorithms, the columns represent various evaluation metrics, and the values in each cell indicate the performance of the corresponding algorithm on a specific metric. Based on the sFC dataset, the best-performing algorithm was GNB, with an accuracy of 0.739, a sensitivity of 0.763, a specificity of 0.719, and an AUC of 0.828.

**Figure 3:**
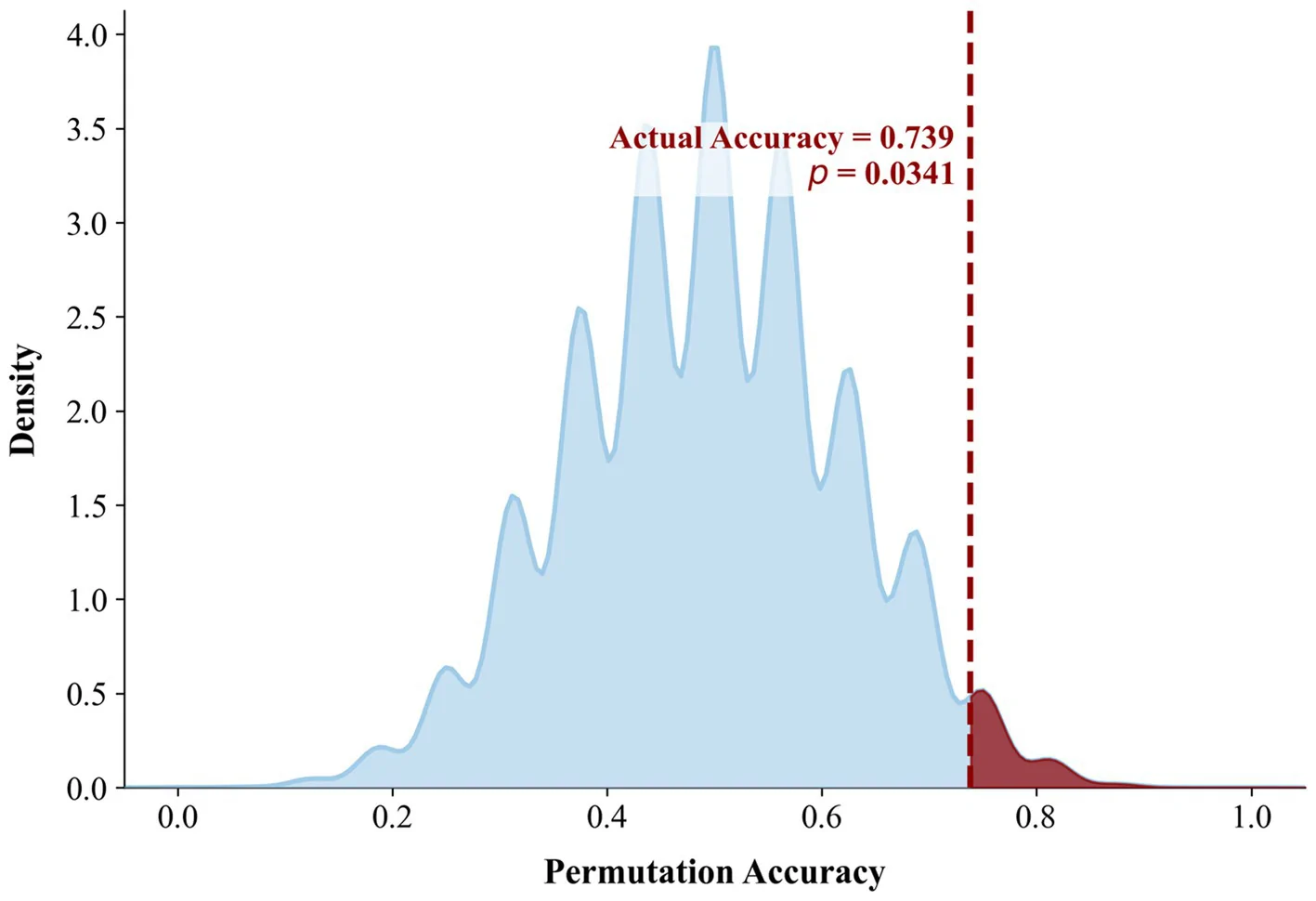
Permutation test results based on sFC.

To verify the statistical significance of the classification results, permutation tests were performed on each dataset, and the best-performing model for each dataset was selected for testing. The class labels of the dataset were randomly permuted, and if the model performance decreased significantly on the permuted data, this indicated that the model had indeed learned meaningful patterns from the data. The distribution and *p* value of the permutation test results for the optimal sFC model are shown in Figure 3. The permutation test showed that the actual classification accuracy of the model using sFC as the input feature was significantly higher than that obtained under randomly assigned labels (*p* < 0.05), indicating that its classification results were statistically significant and that its classification performance was reliable.

**Figure 4:**
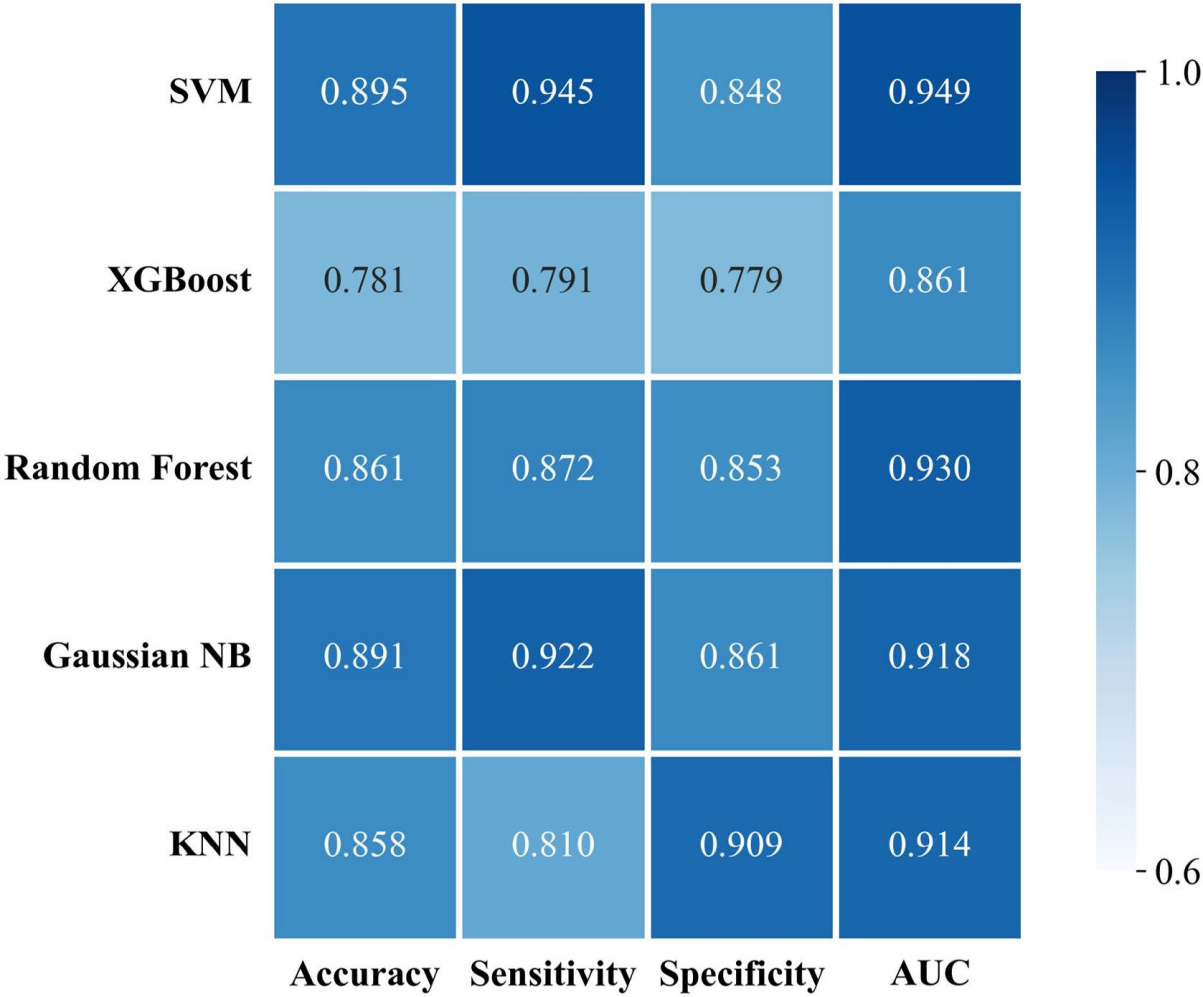
Performance evaluation metrics of different models based on dFC.

The classification results using dFC as features are shown in Figure 4. Based on the dFC dataset, SVM achieved the best performance, with an accuracy of 0.895, a sensitivity of 0.945, a specificity of 0.848, and an AUC of 0.949.

**Figure 5:**
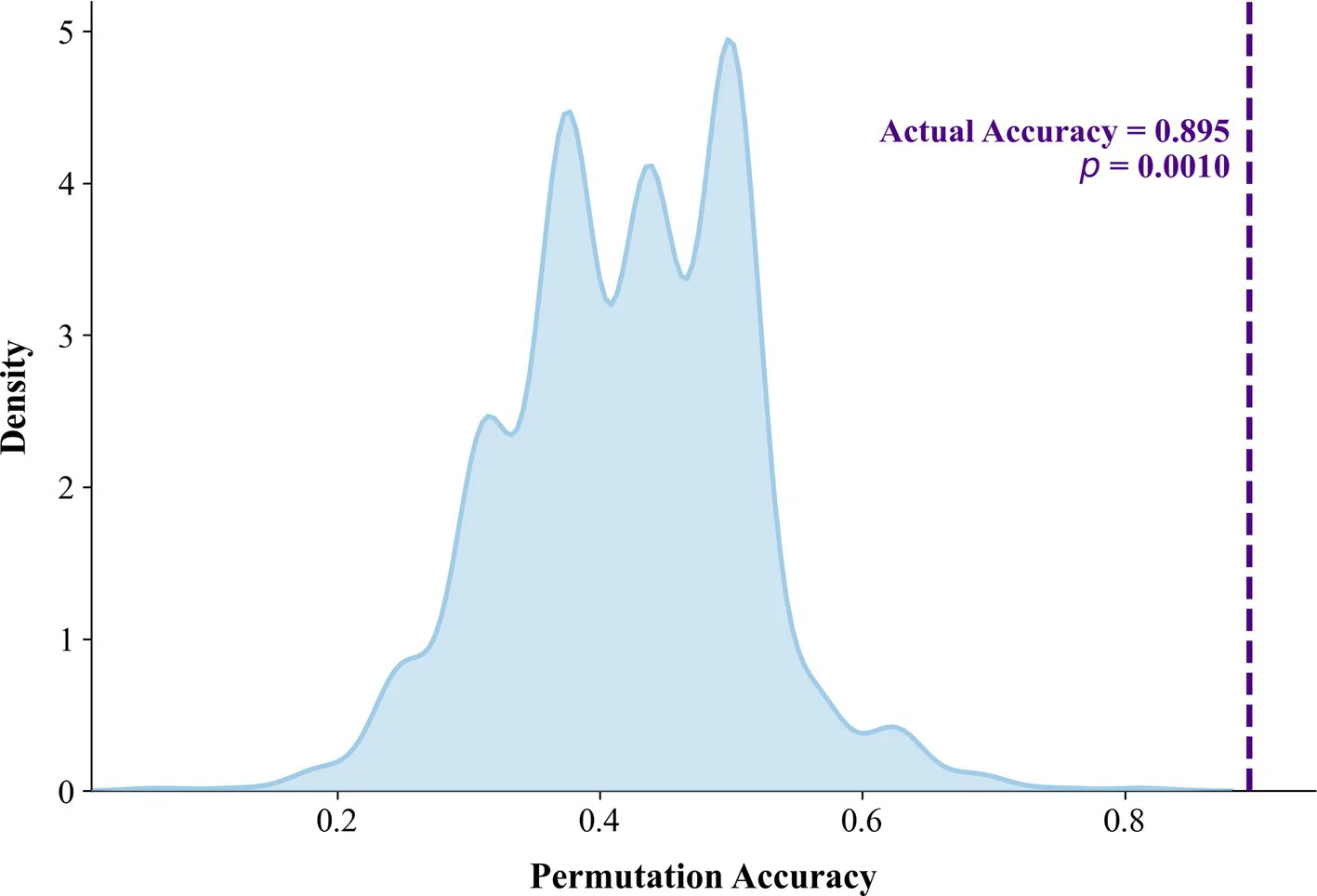
Permutation test results based on dFC.

SVM was selected as the model for the permutation test, and the results are shown in Figure 5. The results indicated that the classification performance of the machine learning model based on the dFC feature dataset was statistically significant (*p* < 0.05).

**Figure 6:**
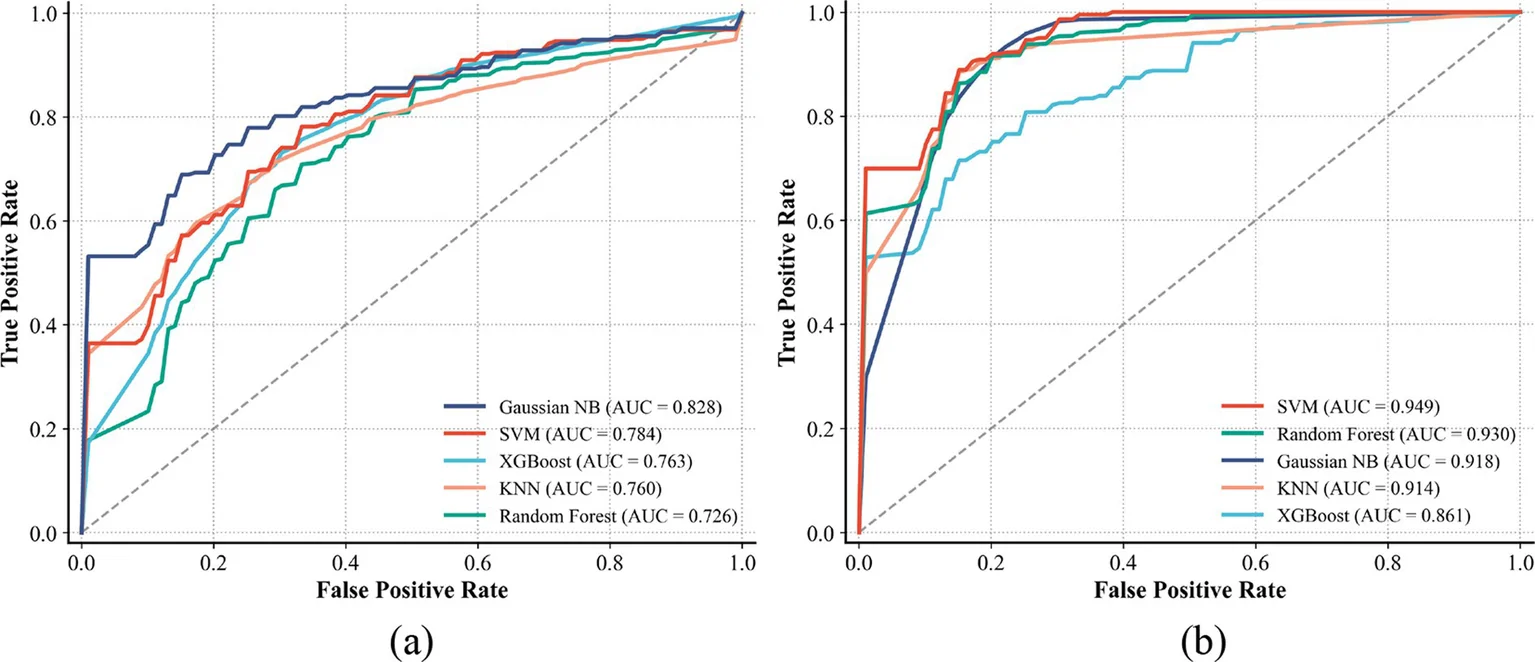
Comparison of ROC curves based on sFC and dFC features. **(a)** ROC curve based on sFC features and **(b)** ROC curve based on dFC features.

The present study systematically evaluated the classification performance of sFC and dFC metrics across multiple ML classifiers. A cross-model comparative analysis showed that ML models using dFC metrics as input features achieved the best classification performance. As shown in Figure 6, the ROC curves provide a clear visual demonstration that the classifiers based on dFC features consistently yielded markedly higher AUC values than their counterparts based on sFC features.

**Figure 7:**
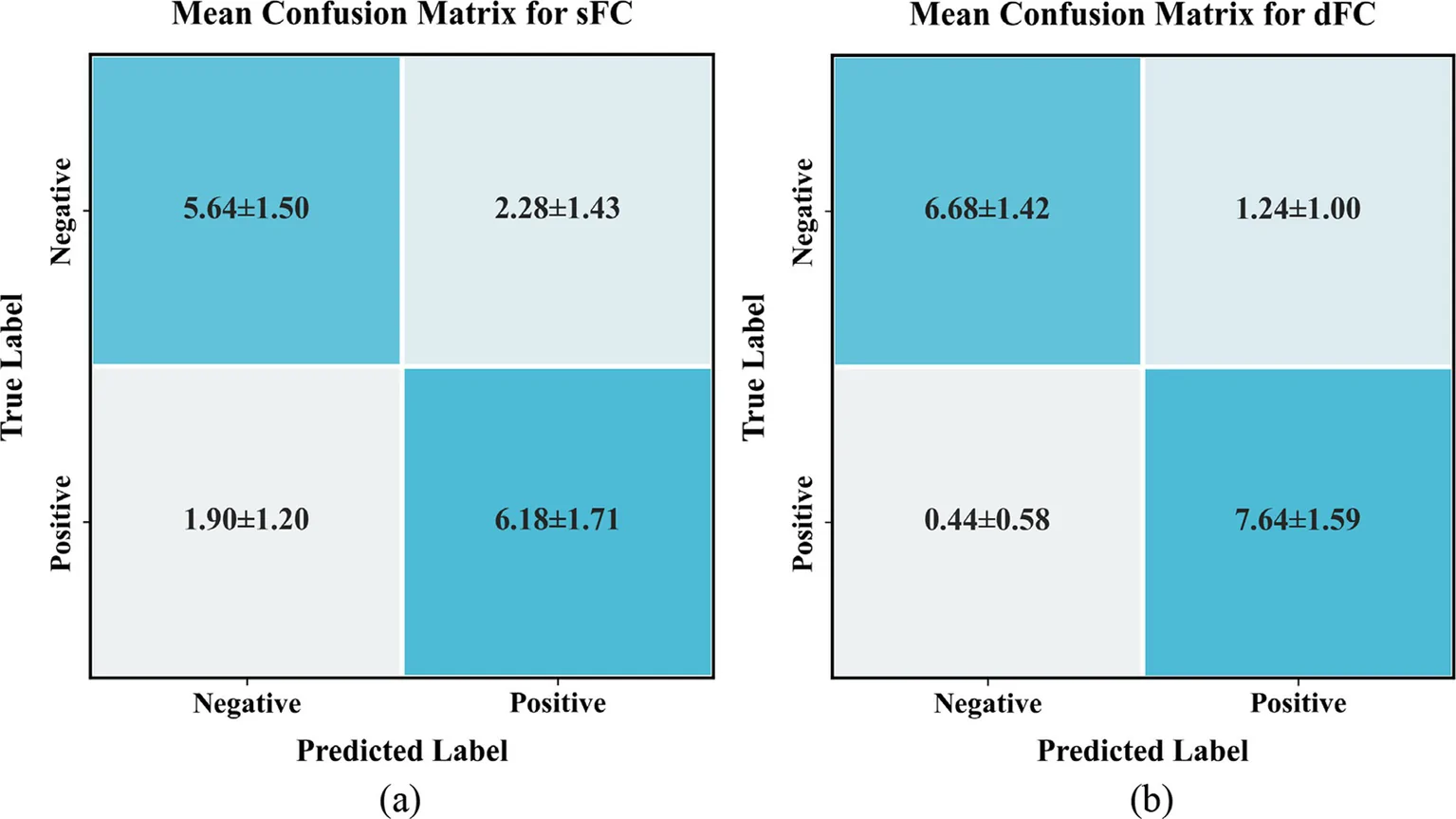
Comparison of the averaged confusion matrices of the optimal models based on sFC and dFC metrics. **(a)** Confusion matrix based on the sFC dataset. **(b)** Confusion matrix based on the dFC dataset.

By calculating the numbers of True Positives (TP), False Positives (FP), True Negatives (TN), and False Negatives (FN), the confusion matrix of each model can be obtained, thereby providing an intuitive quantitative evaluation of classification performance. In the comparison of confusion matrices, the dFC metric showed higher accuracy in identifying both the positive class, comprising flight cadets, and the negative class, comprising ATC students. Specifically, the numbers of TP, FP, TN, and FN were calculated across the 50 independent test evaluations, and an averaged confusion matrix was constructed using the mean ± SD values, as shown in Figure 7.

**Figure 8:**
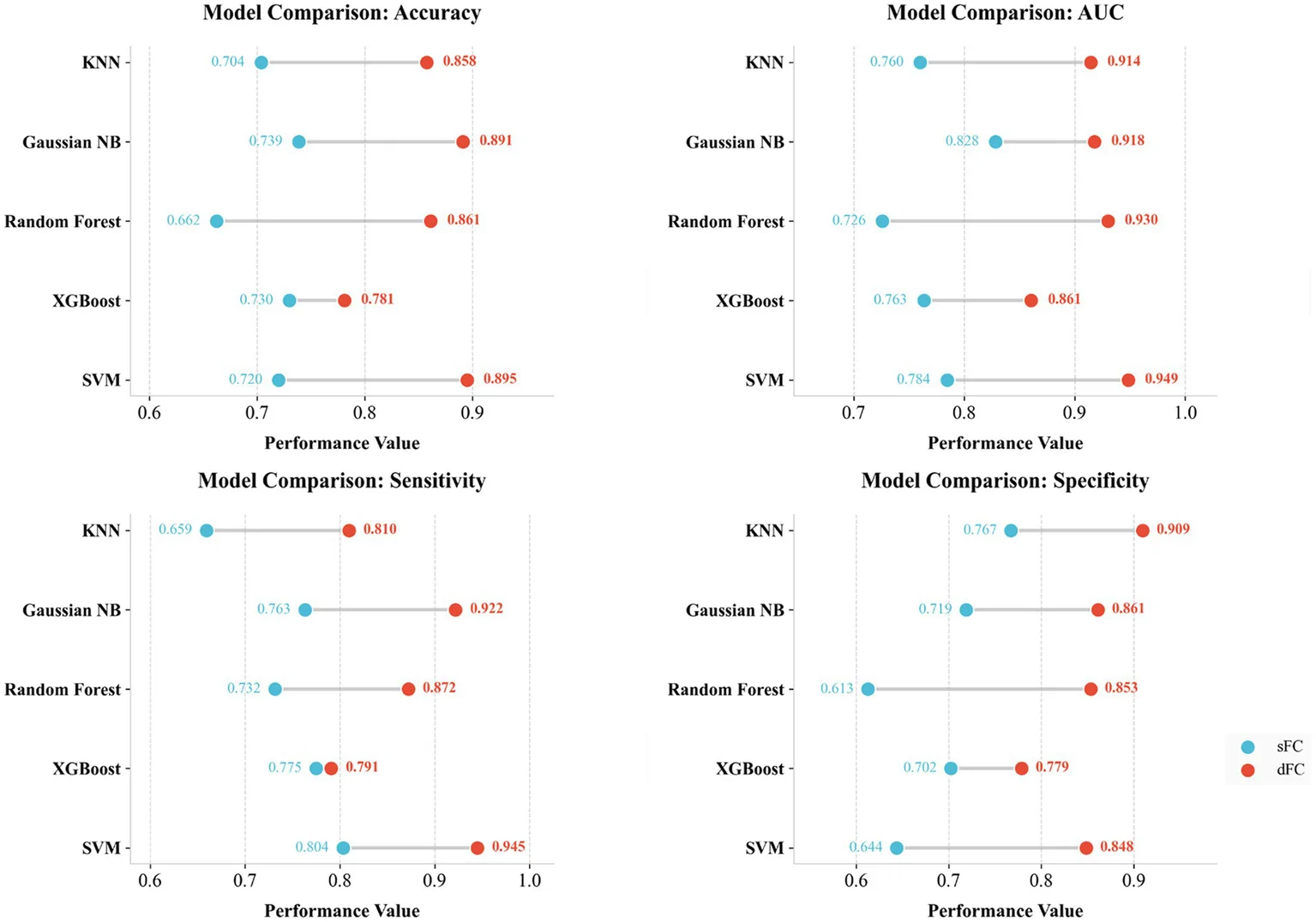
Comparison of classification performance across five models based on sFC and dFC features.

Table 3 summarizes the classification performance of the different ML models using sFC and dFC as input features, and Figure 8 provides a visual presentation of these results. An overall comparison showed that the ML models based on dFC metrics achieved the best classification performance. Among them, the KNN classifier yielded the highest specificity of 0.909, whereas the SVM classifier achieved the highest accuracy, sensitivity, and AUC, reaching 0.895, 0.945, and 0.949, respectively.

**Table 3:** Classification performance based on sFC and dFC features.

Feature set	Model	Accuracy	Sensitivity	Specificity	AUC
sFC	SVM	0.720	0.804	0.644	0.784
	XGBoost	0.730	0.775	0.702	0.763
	RF	0.662	0.732	0.613	0.726
	GNB	0.739	0.763	0.719	0.828
	KNN	0.704	0.659	0.767	0.760
dFC	SVM	0.895	0.945	0.848	0.949
	XGBoost	0.781	0.791	0.779	0.861
	RF	0.861	0.872	0.853	0.930
	GNB	0.891	0.922	0.861	0.918
	KNN	0.858	0.810	0.909	0.914

### Supplementary validation analysis

**Figure 9:**
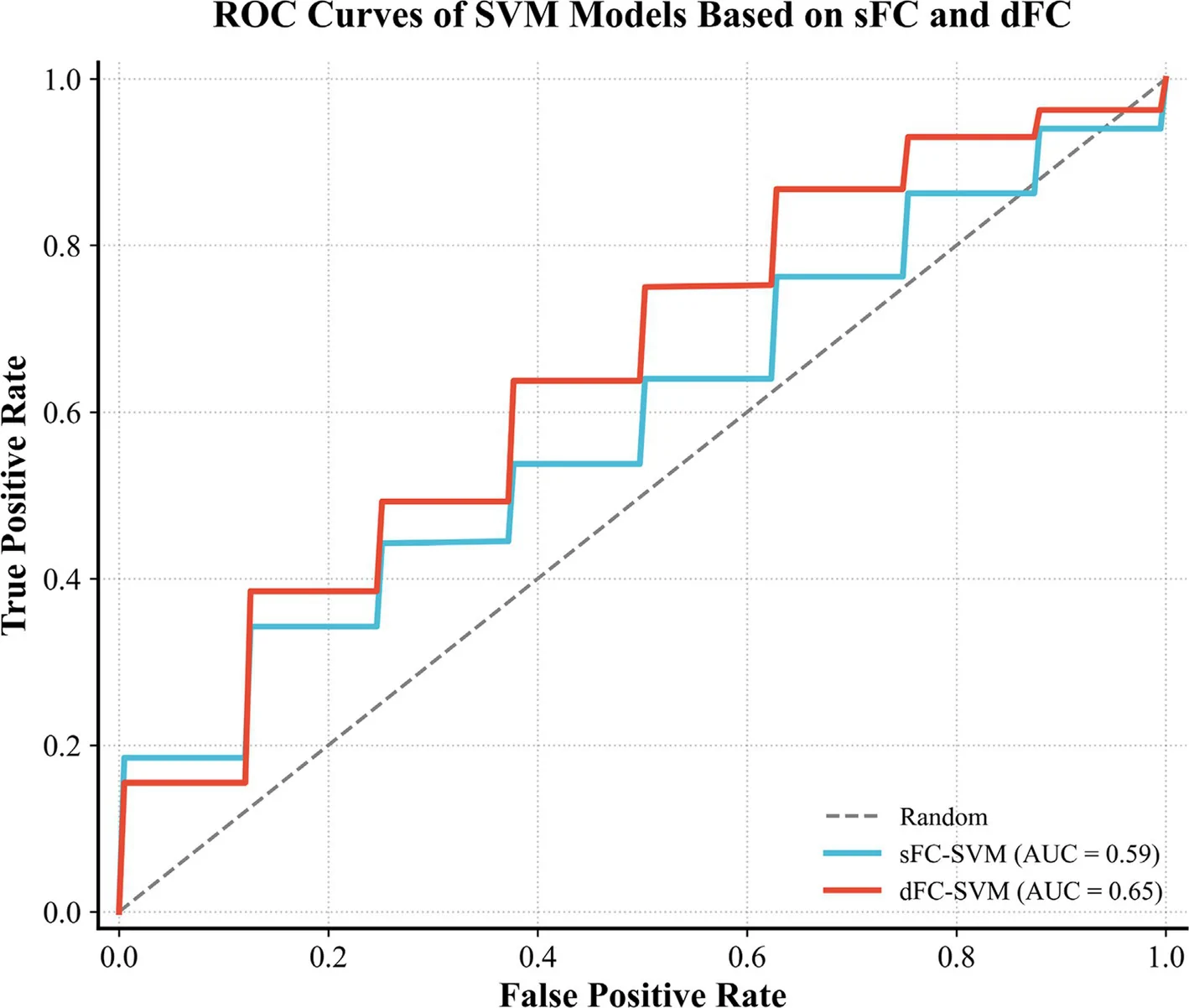
ROC curves of the SVM models based on sFC and dFC metrics.

The results of the supplementary validation analysis are presented in Figure 9, Table 4. Although the classification performance of both models was lower than that observed in the primary analysis, dFC-SVM showed numerically higher values than sFC-SVM across all four evaluation metrics. After excluding the outer test set from the feature-selection process and further controlling feature dimensionality, the performance ranking of dFC relative to sFC did not reverse. No dedicated statistical inference was performed to assess the performance difference between the two models; therefore, these results primarily reflect a trend in the direction of the comparison.

**Table 4:** Comparison of performance metrics for the SVM models based on sFC and dFC metrics.

Performance metrics	sFC	dFC
Accuracy	0.57	0.60
Sensitivity	0.59	0.61
Specificity	0.55	0.59
AUC	0.59	0.65

**Figure 10:**
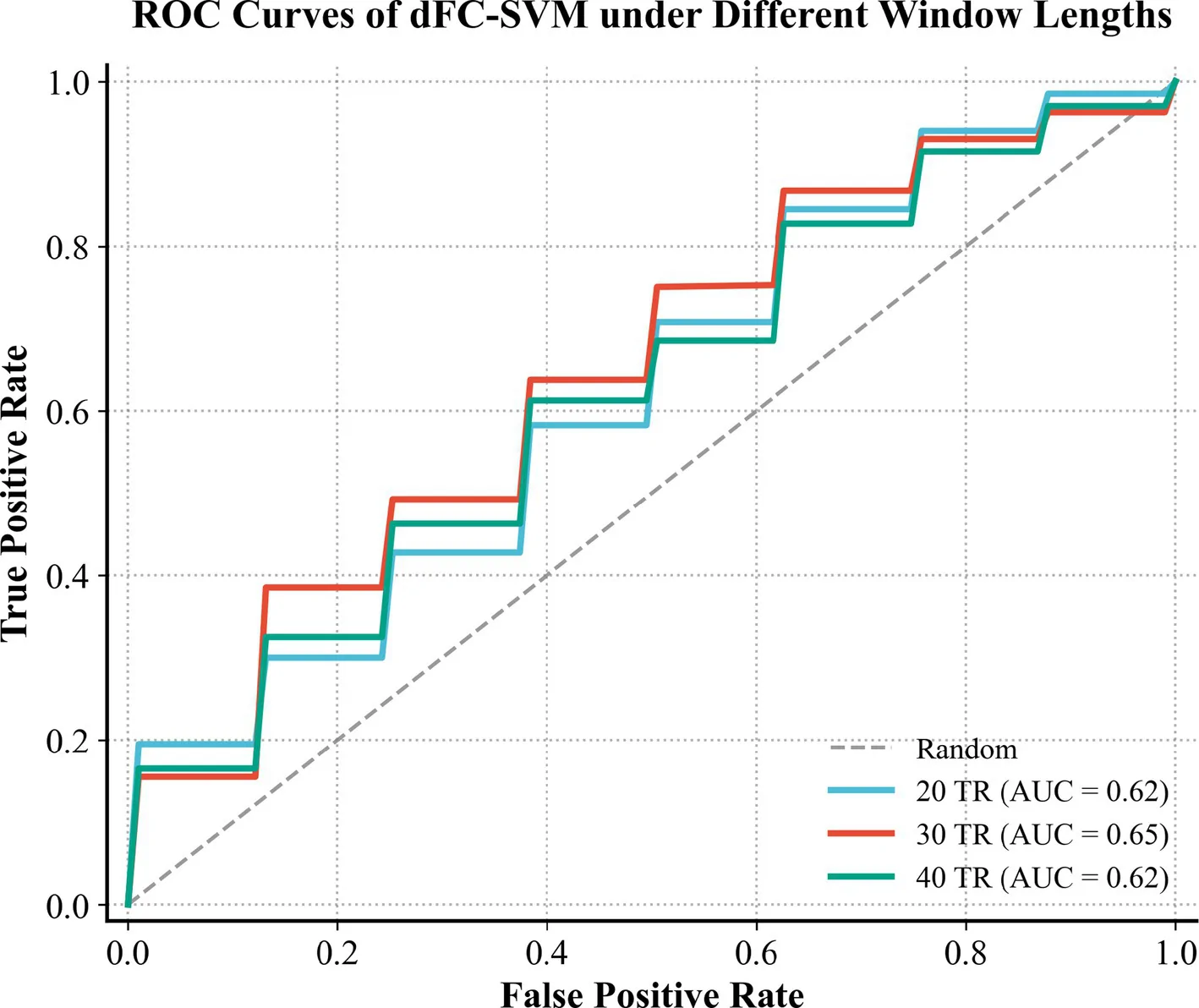
ROC curves of the dFC-SVM models across different sliding-window lengths.

The classification results of the dFC-SVM models across different sliding-window lengths are presented in Figure 10 and Table 5. Overall, model performance remained within a similar range across the three window lengths. The 30 TR window yielded relatively higher values for accuracy, specificity, and AUC, whereas the 40 TR window showed slightly higher sensitivity. The AUC values under all three window conditions exceeded that of the sFC-SVM model obtained using the same supplementary validation procedure (AUC = 0.59), and dFC-SVM performance did not show a marked decline or reversal in the direction of comparison as window length varied. Within the tested range of 20–40 TR, the dFC classification results showed a degree of stability across window lengths, although the specific performance values were still influenced by the choice of window length. These results were primarily intended to evaluate the sensitivity of the model performance pattern to changes in the window parameter.

**Table 5:** Classification performance of the dFC-SVM models across different sliding-window lengths.

Window length	Number of time windows	Accuracy	Sensitivity	Specificity	AUC
20 TR	231	0.57	0.58	0.55	0.62
30 TR	221	0.60	0.61	0.59	0.65
40 TR	211	0.59	0.62	0.57	0.62

### Results of high-contribution features based on the optimal model

**Figure 11:**
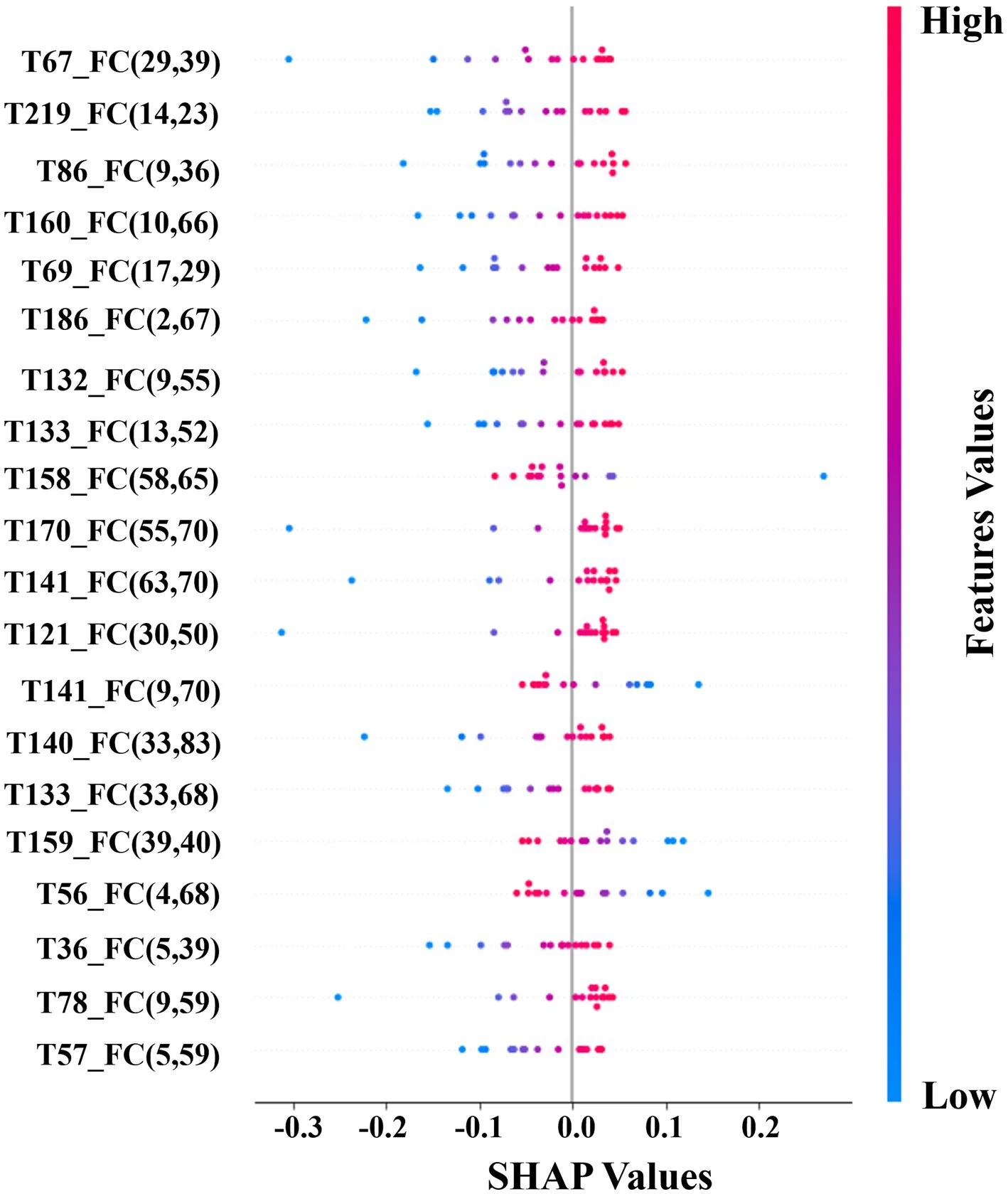
Scatter plot of SHAP values vs. feature values.

Figure 11 presents the 20 FC features that were most important for model prediction. Each row in the figure represents one feature, ordered in descending order according to the sum of its SHAP values, with features ranked higher contributing more to the classification. Each point within a row represents an individual participant. The position of the point on the horizontal axis indicates the magnitude of the SHAP value of that feature for the corresponding participant: a positive SHAP value tends to drive the prediction toward one class (e.g., flight cadets), whereas a negative SHAP value tends to drive the prediction toward the other class (e.g., the control group). The color of each point reflects the magnitude of the feature value for that participant: red indicates a higher strength of the corresponding functional connection, whereas blue indicates a lower connection strength.

As shown clearly in the figure, FC (Ye et al., 2025; Enriquez-Geppert et al., 2024) in time window 67, FC (Xu et al., 2023; Friston, 2011) in time window 219, and FC (Honey et al., 2009; Zhang et al., 2019) in time window 86 were the three most important features for distinguishing the two groups of participants. A detailed analysis of the patterns shown in the figure can further reveal the direction of the influence of changes in specific functional connection strengths on the classification results. Taking the top-ranked feature, FC (Ye et al., 2025; Enriquez-Geppert et al., 2024), as an example, its red points, representing high connection strength, were mainly distributed on the positive side of the SHAP axis, whereas its blue points, representing low connection strength, were mainly distributed on the negative side. This pattern indicates that a higher connection strength of FC (Ye et al., 2025; Enriquez-Geppert et al., 2024) was an important factor driving the model to classify a sample as a flight cadet. Most high-contribution features, such as FC (Pereira et al., 2009; Ye et al., 2025) in time window 69, FC (55, 70) in time window 170, and FC (63, 70) in time window 141, exhibited a similar pattern, suggesting that increased functional connection strength was positively associated with the probability of being predicted as a flight cadet. However, a markedly opposite pattern was also observed in the figure, as exemplified by FC (9, 70) in time window 141. Its red points, indicating high connection strength, were concentrated on the left side (negative SHAP values), whereas its blue points, indicating low connection strength, were concentrated on the right side (positive SHAP values). This complex pattern, in which positive and negative contributions coexist, highlights the distinct roles played by different functional connections in distinguishing the flight cadet group from the control group and further confirms the ability of the machine learning model to capture nonlinear physiological relationships.

**Figure 12:**
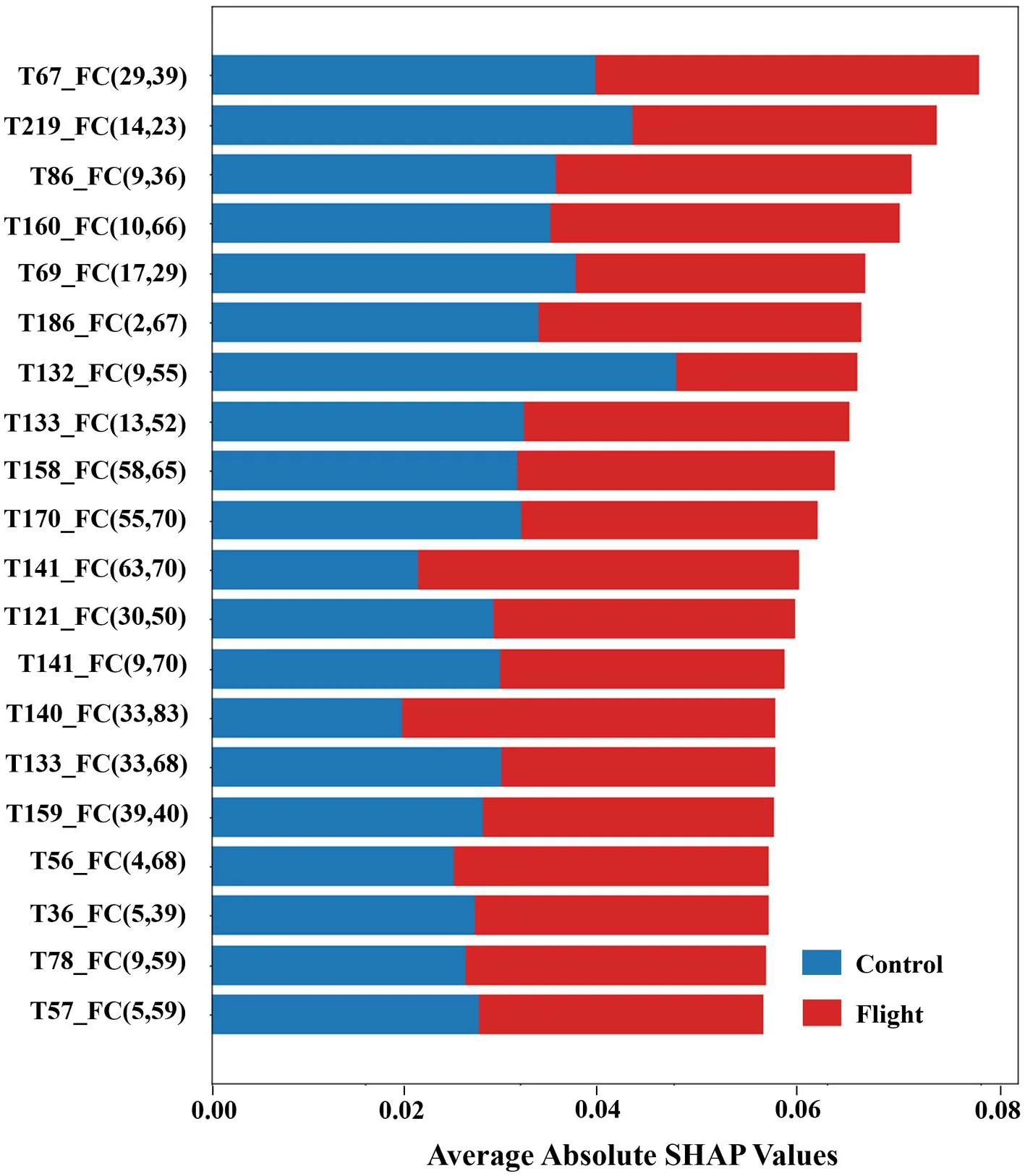
SHAP values of highly contributing features.

Figure 12 presents the top 20 features with the highest contributions, together with the summary of their SHAP values, for the optimal SVM model in distinguishing the flight cadet group from the control group. The vertical axis ranks the features in descending order according to their global importance, whereas the horizontal axis represents the mean absolute SHAP value, which reflects the average magnitude of each feature’s influence on model prediction; longer bars indicate greater contribution. Each bar is composed of stacked blue segments (contribution from the control group) and red segments (contribution from the flight cadet group), thereby revealing the source of each feature’s importance. As shown in the figure, T67_FC (Ye et al., 2025; Enriquez-Geppert et al., 2024), namely the dFC between specific brain regions within a specific time window, was the key feature on which the model relied most heavily for classification. This global importance analysis provides a more comprehensive understanding of the contribution of each feature to the predictive performance of the model.

## Discussion

This study used machine learning methods to construct classification models based on rs-fMRI data and examined brain functional connectivity characteristics associated with actual flight training experience by comparing flight cadets and ATC students with comparable civil aviation educational backgrounds and aviation-related cognitive demands. To investigate the effects of different functional connectivity representations and classification algorithms on model performance, separate sFC- and dFC-based feature sets were constructed and systematically evaluated using five classifiers: SVM, XGBoost, RF, GNB, and KNN. In the primary analysis, the SVM model based on dFC features performed best, with overall classification performance exceeding that of the models based on sFC features. Under a more stringent supplementary validation procedure, the dFC-SVM and sFC-SVM models achieved AUCs of 0.65 and 0.59, respectively. This result was broadly consistent with the direction of comparison observed in the primary analysis and may suggest that dFC contains additional time-varying discriminative information beyond static average connectivity. At the same time, it indicates that model performance was influenced by the timing of feature selection and the high-dimensional feature-processing pipeline. Model performance remained within a similar range across different window lengths, suggesting that the observed direction of the AUC comparison was not driven entirely by the single original window setting of 30 TR, although the specific classification performance remained somewhat sensitive to window length. Spatial localization and large-scale brain network interpretation of the high-contribution features identified in the primary analysis showed that the functional connections associated with group differentiation not only involved sensorimotor and visual information processing supported by the SMN and VN but were also distributed across the DMN, CEN, and VAN, which contribute to internal information integration, cognitive control, attentional switching, goal-directed decision-making, and working memory. The contribution of this study lies not only in comparing the classification performance of sFC and dFC between closely matched occupational groups within the aviation domain, but also in providing a preliminary characterization of neurofunctional features associated with actual flight training experience from the perspective of time-varying coordination among brain networks. These findings provide aviation neuroimaging clues for understanding the neural basis of adaptation to flight training and for future auxiliary assessments incorporating behavioral performance and training outcomes.

Previous studies have demonstrated that classification models based on sFC features can effectively discriminate flight cadets from ordinary college students, with an AUC of 0.83 (Ye et al., 2026). This finding, to some extent, demonstrates the value of sFC features in identifying neural differences between groups. However, recent neuroimaging studies have increasingly found that, because the sFC approach averages signals across the entire scanning period, it may fail to fully reflect the intrinsic temporal dynamics of functional brain activity (Zhang et al., 2019). The brain does not remain in a stable connectivity pattern at all times; rather, it continuously shifts dynamically among different network states. Accordingly, dFC analysis has emerged as an alternative approach that can capture subtle temporal changes in brain network connectivity patterns, thereby providing a richer informational dimension for revealing underlying neural mechanisms (Alonso et al., 2025).

To systematically evaluate the classification performance of sFC and dFC as input features, the present study constructed multiple prediction models based on these two types of features. The experimental results showed that classifiers trained on dFC features generally outperformed models based on sFC. The best-performing dFC-SVM model achieved an AUC as high as 0.949, with an accuracy, sensitivity, and specificity of 0.895, 0.945, and 0.848, respectively (as shown in Figure 4). In contrast, the best-performing model based on sFC features, namely GNB, achieved an AUC of only 0.828, with numerically lower performance metrics than the dFC model in the primary analysis (as shown in Figure 2). This finding may suggest that dFC captured additional time-varying information relevant to distinguishing flight cadets from ATC students. One possible explanation for the numerically better performance of the dFC-based model in the primary analysis is that, during rs-fMRI scanning, although participants were instructed to keep their eyes closed, remain relaxed, and avoid deliberate thinking, uncontrollable spontaneous cognitive fluctuations may still occur, such as brief attentional lapses or emotional fluctuations. These transient intrinsic mental activities may cause brain functional connectivity to exhibit rapid and complex patterns of change across time, and such changes constitute the core information that dFC analysis is designed to capture (Alonso et al., 2025). Because sFC relies on a whole-session averaging strategy, it may obscure these transient fluctuations and thereby reduce its ability to capture latent state-related information (Lemm et al., 2011). In the specific context of the present study, such dynamic changes may contain discriminative information for distinguishing flight cadets from ATC students. Owing to prolonged exposure to intensive training, flight cadets may have undergone systematic enhancement in executive function, attentional regulation, spatial orientation, and cognitive flexibility (Enriquez-Geppert et al., 2024). The neural basis of these higher-order cognitive abilities may be reflected more strongly in how relevant brain networks, such as DMN and CEN, dynamically couple and switch in an efficient and flexible manner (Ye et al., 2025), rather than in the mean strength of their static connectivity (Martínez et al., 2018). By means of the sliding-window technique, dFC can track the temporal evolution of connection strength within these brain networks, providing complementary information for exploring dynamic brain network characteristics associated with flight training experience. Thus, dFC provides time-varying information that is not captured by whole-session sFC and showed a numerical performance advantage in the present classification analyses. This pattern suggests that dFC may serve as a complementary representation for investigating complex cognitive abilities and neurofunctional characteristics associated with flight training experience, although further validation is required.

The supplementary validation provided a more conservative reference for interpreting the findings of the primary analysis. When the t-test was restricted to each outer training set and PCA was further incorporated into the training pipeline to control feature dimensionality, the sFC-SVM and dFC-SVM models achieved AUCs of 0.59 and 0.65, respectively. Although the performance of both models was lower than that observed in the primary analysis, dFC-SVM retained a numerical advantage over sFC-SVM in accuracy, sensitivity, specificity, and AUC. This result was broadly consistent with the direction of comparison observed in the primary analysis, suggesting that the temporal information contained in dFC may provide some additional discriminative information for distinguishing flight cadets from ATC students. It should be noted, however, that the magnitude of the advantage of dFC over sFC was limited in the supplementary validation. The relatively high classification performance observed in the primary analysis may have been influenced, at least in part, by the timing of feature selection, the high-dimensional feature structure, and the dimensionality-reduction strategy and should not be interpreted directly as evidence of stable and generalizable individual-level predictive ability. Because the supplementary validation simultaneously modified both the timing of feature selection and the dimensionality-reduction procedure, the change in performance cannot be attributed to either component alone. Taken together, the advantage of dFC over sFC is more appropriately interpreted as a comparative trend that requires further validation, whereas the high-contribution connections identified in the primary analysis should be regarded as candidate brain network correlates associated with flight training experience rather than independently validated and stable biomarkers.

Considering the performance of the five different classification algorithms in handling dFC and sFC features, it can be seen that there is a close interaction between the choice of algorithm and the intrinsic properties of the data features. No single classifier is absolutely optimal under all conditions; rather, each has its own adaptability, advantages, and limitations.

Sliding-window length is an important parameter affecting dFC estimation. Shorter windows can preserve relatively rapid connectivity changes and provide higher temporal resolution, but the smaller number of time points available for estimating correlations within each window makes the estimates more susceptible to noise and limited sampling. Longer windows may improve the stability of functional connectivity estimation, but they may also smooth connectivity changes of shorter duration (Hutchison et al., 2013; Shakil et al., 2016). The present study therefore further compared window lengths of 20, 30, and 40 TR within the same supplementary validation framework. The dFC-SVM models achieved AUCs of 0.62, 0.65, and 0.62 under the three conditions, respectively. These values were generally within a similar range and were all higher than the AUC of 0.59 obtained for sFC-SVM. This may suggest that the numerical AUC advantage of dFC over sFC in the supplementary validation was not driven entirely by the single original window setting of 30 TR. Among the three conditions, the 30-TR window yielded relatively higher accuracy, specificity, and AUC, possibly reflecting a more appropriate balance between temporal resolution and the stability of within-window correlation estimates in the current dataset. Because the evaluation metrics still showed some variation across window lengths, these findings are more appropriately interpreted as indicating that the main direction of comparison showed a degree of stability within the tested range of 20–40 TR.

In the primary analysis, SVM yielded the highest performance among the evaluated classifiers when applied to dFC features. The dFC-based SVM model achieved the highest overall AUC (0.949) and sensitivity (0.945), indicating a certain capacity to learn high-dimensional discriminative patterns under the current dataset and feature-processing pipeline (Vapnik, 1999). Neuroimaging data are typically characterized by small sample sizes and high dimensionality. Through its maximum-margin principle and kernel trick, SVM can effectively mitigate the curse of dimensionality and overfitting, thereby enabling the precise capture of subtle patterns that distinguish between groups (Orrù et al., 2012). However, when applied to the relatively less informative sFC features, although SVM maintained the highest sensitivity (0.804), its specificity dropped to the lowest level among all models (0.644), indicating a tendency to favor the prediction of positive samples. This suggests that when feature information is insufficient, SVM may sacrifice performance on one class in order to maximize recognition of the other, thereby leading to an imbalance in model performance (He and Garcia, 2009).

In contrast to SVM, the GNB classifier exhibited a different performance profile. GNB showed stable performance in the dFC task, ranking closely behind SVM, and emerged as the best overall model in the sFC task (AUC = 0.828). The structure of the GNB model is relatively simple. Although its core assumption of conditional independence among features is difficult to fully satisfy in functional connectivity data, this simplification may instead become an advantage (Arbabshirani et al., 2013). For sFC data with relatively limited discriminative power, more complex models, such as RF, may be more prone to learning noise and thus overfitting, whereas the simple probabilistic generative model of GNB may exhibit greater robustness. Its favorable performance suggests that even a simple linear classifier may outperform more complex nonlinear models when feature quality becomes the principal bottleneck (Varoquaux, 2018).

The decision tree-based ensemble models, namely RF and XGBoost, performed poorly in the present study. In the dFC task, XGBoost ranked lowest across all performance metrics, whereas in the sFC task, RF likewise showed the worst performance among all models. One possible explanation for this phenomenon lies in the use of the sliding time-window approach for dFC in the present study, in which the window size was determined empirically. The use of sliding windows may cause adjacent connectivity matrices to exhibit a certain degree of similarity, thereby increasing collinearity at the feature level. When tree-based models perform node splitting, a high degree of collinearity among features may lead to instability in the construction of individual decision trees, which in turn may affect the final performance of the entire forest (Strobl et al., 2008). In addition, these two types of models involve numerous hyperparameters that require careful tuning. Their full potential may therefore not have been completely exploited in the present study, resulting in performance inferior to that of models with simpler structures or lower sensitivity to hyperparameter settings.

KNN exhibited a distinctive performance pattern in both the dFC and sFC tasks: it consistently achieved the highest specificity among all models, while simultaneously showing relatively low sensitivity. As an instance-based lazy learning algorithm, the performance of KNN directly reflects the geometric distribution of the data in feature space (Liaw et al., 2010). The persistently high specificity suggests that the brain functional connectivity patterns of ATC students, serving as the control group or negative samples, formed a relatively compact and homogeneous cluster in feature space, such that most of their neighboring samples belonged to the same class. In contrast, the lower sensitivity implies that the connectivity patterns of flight cadets, as the positive samples, may have been more heterogeneous and more diffusely distributed, with possible overlap at the boundary with the control group, thereby making it more likely for samples from this group to be surrounded by neighbors from a different class. The performance profile of KNN not only evaluated classification effectiveness, but also provided valuable clues for understanding the intrinsic distributional characteristics of neural features in different groups.

In the optimal model, the connection between the left insula and the left parahippocampal gyrus accounted for a high proportion of the feature weight. The left insula is a key cortical region involved in interoception and emotional awareness (Craig, 2009). The left parahippocampal gyrus, particularly the parahippocampal place area, is crucial for the perception and recognition of visual environments (Epstein and Kanwisher, 1998). In addition, other highly weighted connections in the model involved brain regions such as the right inferior frontal gyrus, triangular part; the left medial superior frontal gyrus; the right posterior cingulate cortex; the orbital part of the bilateral middle frontal gyrus; and the right angular gyrus. The right inferior frontal gyrus, triangular part, is widely regarded as a key node for response inhibition and forms part of the brain’s cognitive control system (Aron et al., 2014). The left medial superior frontal gyrus is a key component of the neural network underlying working memory, particularly during tasks requiring higher-level executive processing (de Boisgueheneuc et al., 2006). The right posterior cingulate cortex plays a critical role in regulating the attentional balance between internally directed cognition and externally focused tasks (Leech and Sharp, 2013). the orbital part of the middle frontal gyrus, located within the orbitofrontal region, is involved in emotion regulation (Cao et al., 2023). The right angular gyrus serves as a crucial transmodal hub, where multiple types of sensory information converge and are integrated to support event comprehension and meaning attribution (Seghier, 2013). Taken together, these highly weighted brain regions form a complex network involving internal state monitoring, spatial navigation, executive control, and multisensory information integration.

**Figure 13:**
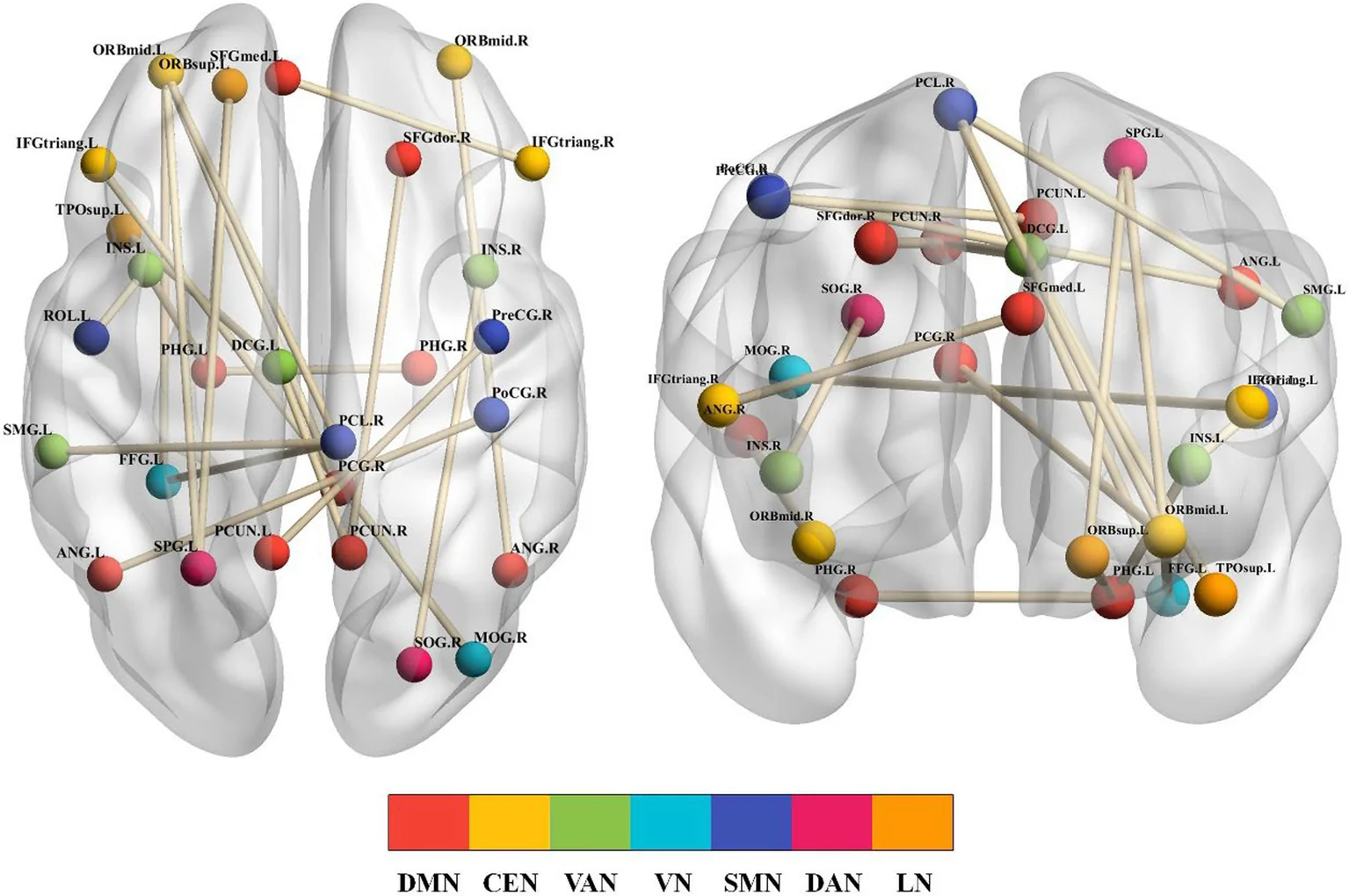
Spatial distribution and associated brain networks of high-contribution features.

According to the seven-network template developed by Yeo et al. (2011) the 20 FC features that were most important for model prediction were assigned to seven brain networks, as shown in Figure 13. The results indicated that DMN accounted for the largest proportion of the overall weight. CEN, the Ventral Attention Network (VAN), SMN, and the Visual Network (VN) also accounted for relatively large proportions, whereas the Dorsal Attention Network (DAN) and the Limbic Network (LN) contributed relatively little weight.

These network-level classification results provide a more macroscopic perspective for understanding the neural basis of cognitive abilities in flight cadets. DMN accounted for the largest proportion of weight in the model. The primary functions of DMN involve self-referential processing, episodic memory retrieval, and simulation of future events (Buckner et al., 2008). In a high-load and high-risk task such as flying, success depends not only on immediate responses to the external environment, but also on whether pilots can effectively utilize internal cognitive resources, such as recalling training procedures and planning subsequent operations. An efficient DMN may reflect a stronger capacity for internal information integration and mental simulation in cadets even during the resting state, which is crucial for coping with complex flight situations. CEN and VAN also accounted for substantial weights. CEN is a task-positive network that governs working memory, cognitive control, and goal-directed decision-making (Seeley et al., 2007), whereas VAN is a bottom-up attentional system responsible for rapidly reorienting attention toward salient stimuli in the environment (Corbetta and Shulman, 2002). Notably, brain regions identified in the present study, such as the bilateral insula, are core nodes of SN, which serves as a central hub for attentional switching (Molnar-Szakacs and Uddin, 2022). SN and VAN show a high degree of anatomical and functional overlap and coordination (Zhang et al., 2025), and SN may even be regarded as a key component within VAN that is responsible for detection and initiation (Corbetta and Shulman, 2002; Menon and Uddin, 2010). The core function of SN lies in integrating interoceptive information with external sensory input in order to rapidly identify which events are most important for the individual (Zhang et al., 2025), after which VAN is recruited to execute attentional reorientation (Corbetta and Shulman, 2002). At a deeper level, the findings of the present study suggest that a brain capable of efficiently predicting flight performance requires not only a sensitive attentional system (VAN), but, more importantly, depends on its core component, SN, to make accurate judgments and rapid responses. The contributions of VN and SMN directly reflect the nature of flight tasks, namely, performing precise bodily perception and manual operations on the basis of continuous visual information, such as instrument displays and weather conditions. The importance of these two networks is consistent with the functions of brain regions identified in this study, such as the left parahippocampal gyrus, which is involved in visual scene recognition, and together they form the neural basis for cadets’ perception and control of the aircraft.

Our previous study (Ye et al., 2025) found that, compared with controls without flight experience, flight cadets exhibited enhanced functional connectivity between the CEN and DMN in a specific dynamic state, together with increased mean dwell time and fractional windows in that state, whereas no significant between-group difference was observed in the transition number. Chen et al. (2020), however, found that active-duty civil aviation pilots with greater flight experience, compared with healthy controls, exhibited not only enhanced connectivity between the CEN and DMN, but also enhanced connectivity between the CEN and SN, reduced within-CEN connectivity, and more frequent transitions between brain states. Taken together, these two studies may suggest that coordination between the CEN and DMN represents a relatively consistent resting-state network characteristic of individuals with flight experience compared with those without flight experience. The more extensive reorganization of the triple networks and differences in state-transition dynamics observed in active-duty pilots may, in turn, be related to their longer-term professional flight experience. In the present study, connections involving the DMN, CEN, and the insula-related VAN and SN made relatively high contributions to model classification, showing a certain degree of network-level consistency with the findings described above. It should be emphasized that the study by Ye et al. (2025) used the same participant cohort as the present study, whereas the study by Chen et al. (2020) differed from the present study in participant age, flight hours, control-group type, and analytical procedures. The available cross-study evidence remains insufficient to establish a stage-related trajectory of brain network changes across healthy individuals without flight experience, flight cadets, and experienced pilots. The above interpretation may therefore serve as a hypothesis for future studies directly comparing these three groups under a unified study design.

From the perspective of aviation training and assessment, existing flight cadet training systems rely primarily on aeromedical fitness requirements, stage-based evaluations, licensing training, and actual operational performance. Although these indicators play an irreplaceable role in ensuring flight safety, they mainly reflect individuals’ observable health status and behavioral performance and are less able to directly characterize intrinsic differences in brain functional connectivity patterns and their dynamic regulation. The dFC features identified in this study provide candidate neuroimaging clues for investigating time-varying coordination between the visual and sensorimotor systems and higher-order cognitive control networks in flight cadets. These findings may help elucidate, at the brain network level, neural adaptations associated with flight training experience and provide a complementary perspective to existing flight training evaluation systems that predominantly rely on external performance measures. Although the present study yielded meaningful findings in the quantitative identification of flight cadets and the exploration of underlying brain network mechanisms, several limitations remain and should be addressed in future research. First, there is the inherent limitation of the cross-sectional study design. Although this study examined significant differences in the dynamic characteristics of functional brain networks, particularly the triple-network architecture, between flight cadets and ATC students, it cannot establish an absolute causal relationship between flight training and dynamic alterations in brain networks. Future longitudinal studies are needed to determine whether the adaptive characteristics of these higher-order cognitive networks are shaped by long-term intensive flight training or instead reflect intrinsic biological advantages that are already present at the early selection stage. Second, the selection of the control group has certain limitations. Although the use of ATC students as the control group helped to control, to some extent, for factors such as institutional environment, years of education, civil aviation-related professional education, and the cognitive task characteristics associated with aviation operations, ATC students cannot be regarded as a fully matched control group for flight cadets. Flight cadets are additionally required to undergo aviation occupation-related aeromedical evaluations, license-oriented flight training, and stage-specific assessments during both admission and subsequent training. Meanwhile, ATC training also involves dynamic information monitoring, attentional allocation, and other cognitively demanding processes. Future studies could adopt a unified analytical framework and simultaneously include active-duty pilots with varying levels of professional flight experience, flight cadets at different stages of training, ATC students, and other general non-flying individuals to further disentangle the potential effects of professional flight experience, flight training, civil aviation educational background, and occupational selection. Third, the sample size and high-dimensional feature-processing pipeline may have limited the stability of the model performance estimates. The present study included 76 participants, and both the sFC and dFC data exhibited the typical characteristics of a small-sample, high-dimensional setting. In particular, the unfolded dFC features comprised a large number of correlated functional connections across temporal windows. Moreover, the *t*-test was performed before the data were divided into training and test sets, introducing the possibility that the classification performance obtained in the primary analysis was optimistically biased. The supplementary validation results provided preliminary support for the possibility that dFC contains additional time-varying discriminative information, while also indicating that model performance and feature interpretation were highly sensitive to the analytical pipeline. Future studies should increase the sample size and adopt a more rigorous nested validation framework in independent datasets, with feature selection, data scaling, dimensionality reduction, and hyperparameter optimization all restricted to the corresponding training folds, to further assess model generalizability and feature stability. Fourth, the range of parameter validation for dynamic functional connectivity remained limited. In addition to the original 30 TR window, the present study compared window lengths of 20 and 40 TR. Model performance remained generally within a similar range across the three conditions, providing preliminary support for the stability of the main direction of comparison across window lengths. However, this analysis covered only three fixed window lengths within the range of 20–40 TR, all using the same sliding step of 1 TR and a fixed rectangular window. The effects of window step size, window shape, and different frequency-processing strategies on dFC estimation were therefore not systematically evaluated. The use of a fixed window width inevitably leads to a high degree of similarity between adjacent connectivity matrices, thereby introducing multicollinearity at the feature level, which may also have contributed to some extent to the instability of tree-based models during node splitting. Subsequent studies should systematically evaluate the effects of different temporal scales, including multiple window lengths and step sizes, in order to more comprehensively characterize the evolutionary trajectory of dynamic switching in brain networks. Fifith, the feature modality was limited to a single source. The present study was based solely on rs-fMRI. Future research may consider integrating multimodal physiological signals, such as electrocardiography, electrodermal activity, and other polyphysiological data, together with task-based fMRI and behavioral scales, to construct multidimensional feature-fusion models. Such efforts may provide more comprehensive, precise, and quantifiable objective physiological indicators for the competency assessment and evaluation system of pilots as a special occupational group. Sixth, the present study included only male flight cadets. Although this criterion helped to reduce potential sex-related confounding effects and improve sample homogeneity, it also limits the generalizability of the findings to female flight cadets or female aviation professionals. Future studies with larger samples and a more balanced sex distribution are needed to verify the stability and generalizability of the present findings.

## Conclusion

Using ATC students with comparable civil aviation educational backgrounds and aviation-related cognitive demands, but no actual flight experience, as the control group, this study systematically compared the performance of static and dynamic functional connectivity in distinguishing flight cadets from ATC students. Five machine learning algorithms and interpretability analyses were further applied to investigate time-varying brain network characteristics associated with actual flight training experience. In the primary analysis, the SVM model based on dFC features achieved the best performance. Under a more stringent supplementary validation procedure, dFC-SVM showed better performance than sFC-SVM. Results obtained across different window lengths further suggested that dFC may contain additional time-varying discriminative information beyond static average connectivity. Overall, the present findings are more appropriately interpreted as preliminary evidence that dFC may provide additional discriminative value relative to sFC. The high-contribution connections identified in the primary analysis mainly involved regions including the left insula, left parahippocampal gyrus, and right triangular part of the inferior frontal gyrus and were widely distributed across networks such as the DMN, CEN, VAN, SMN, and VN. These findings may suggest that neurofunctional differences between flight cadets and ATC students involve not only brain networks supporting sensorimotor and visual information processing, but also the dynamic coordination of cognitive control under high-pressure and rapidly changing conditions. Compared with previous studies using ordinary university students as controls, the use of a comparable ATC student group within the aviation domain may help control, to some extent, for general civil aviation educational background and aviation-related cognitive demands, thereby allowing a more targeted characterisation of neural features associated with actual flight training experience. These findings provide aviation neuroimaging clues for future longitudinal and independent-sample validation incorporating training stage, behavioral performance, and actual flight performance, as well as for exploring adaptive monitoring of flight training and personalised multidimensional auxiliary assessment.

## Data Availability

The raw MRI data are not publicly available due to legal restrictions, as the sharing of physiological data from pilots is prohibited by the Civil Aviation Administration of China. Requests for other research materials, including sFC- and dFC-derived feature data and machine learning analysis code, may be directed to the corresponding author at: yandongfeng@cafuc.edu.cn.

## Footnotes

1
https://pubmed.ncbi.nlm.nih.gov/36659803/

2
https://guorongwu.github.io/DynamicBC/
